# Emotional labor as emotion regulation investigated with ecological momentary assessment – a scoping review

**DOI:** 10.1186/s40359-023-01469-9

**Published:** 2024-02-12

**Authors:** Corinna Pinkawa, Denise Dörfel

**Affiliations:** 1 Faculty of Psychology, Ruhr University Bochum, Bochum, Germany; 2 Faculty of Psychology, Work- and Organizational Psychology, TUD Dresden University of Technology, Dresden, Germany

**Keywords:** Emotional Labor, Emotion Regulation, Ecological Momentary Assessment, Ambulatory Assessment, Experience Sampling, Stress, Strain, Work, Job

## Abstract

**Background:**

This scoping review’s aim is to provide a comprehensive overview of ecological momentary assessment (EMA)- based research on emotional labor (EL) as emotion regulation (ER). This includes an examination of the theoretical foundation this research builds on, how the hypothesized relationships are investigated using EMA methods as well as the studies’ findings themselves. We built on the work of Grandey and Melloy (J Occup Health Psychol 22:407–22, 2004), who broadly distinguished between the two regulatory strategies of deep acting (DA) and surface acting (SA), embedded in a hierarchical model of emotional labor, as a guiding theory for structuring this review.

**Methods:**

To be included, studies had to use EMA to measure SA or DA, with no restrictions regarding population and date of publication. The electronic databases CINAHL, PsycArticles, PsycINFO, PSYNDEX, Embase, PubMed, and Web of Science were searched. Studies were included if they met the above criteria and were written in English or German. Out of 237 publications, 12 were chosen for this review.

**Results:**

All studies were based on emotional labor theories, with Arlie Hochschild’s theory being the most popular, followed by Alicia Grandey's emotional labor theory and its modifications (Grandey AA. Emotion Regulation in the Workplace: A New Way to Conceptualize Emotional Labor; Grandey AA. When “the show must go on”: Surface acting and deep acting as determinants of emotional exhaustion and peer-rated service delivery. 2003). The methodological quality of the studies varied greatly. The results of the studies indicate that SA is influenced by negative events, trait SA, service innovation and certain emotions, while DA is influenced by positive events and emotional intelligence. Emotional labor benefits the organization, e.g., by improving customer conflict handling, but also causes harm by increasing employee withdrawal behavior. For the employee, emotional labor results in more harm than benefits.

**Conclusions:**

The research area is still in its early stages and the findings are mostly consistent, but the small number of studies needs to be replicated to increase the reliability of the results. The lack of evidence for ertain hypotheses highlights the presence of unresolved relationships that require further exploration. We are only at the beginning of investigating emotional labor using ecological momentary assessment, and conducting more high-quality studies will significantly enhance our comprehension of emotional labor.

**Supplementary Information:**

The online version contains supplementary material available at 10.1186/s40359-023-01469-9.

## Background

Emotional labor has been extensively studied as a psychosocial topic in relation to work-related stress over the past few decades [1]. Work-related stress can be defined as "the emotional, cognitive, behavioral and physiological reaction to aversive and noxious aspects of work, work environments and work organizations." [2]. Similarly, emotions are responses to internal or external stimuli that are perceived as relevant to the individual and characterized by a subjective experience, central and peripheral physiology, and particular behavior [3]. Workplace stressors represent significant stimuli that can increase the likelihood of stress reactions, including negative emotional experiences, as outlined in the Affective Events Theory by Weiss and Cropanzano [4]. The attitudinal and behavioral consequences of emotional experiences can impact work performance, job satisfaction, and withdrawal behaviors such as turnover and absenteeism. Workplace stressors arise from several organizational conditions, ranging from workload and job control to social factors such as the existence of display rules [5–7].

Around the turn of the millennium, during the so-called *affective revolution* in psychology and organizational behavior, emotional labor gained significant attention as a method to deal with the escalating interpersonal demands of work [8]. Coping is defined as the "constantly changing cognitive and behavioral efforts to manage specific external and/or internal demands that are appraised as taxing or exceeding the resources of the person" [9]. Emotional labor involves implementing effortful strategies (i.e., emotion regulation, [10] to meet explicit (i.e., display rules) or implicit emotional requirements (e.g., interaction expectations, work motives) with the aim of achieving organizational goals [8, 11, 12]. Emotional labor and emotion regulation extend beyond the concept of coping because both processes not only take place in negative or challenging scenarios. For instance, emotion regulation and emotional labor may lead to negative emotions when they are deemed appropriate in a specific situation or profession [13–15]. In the funeral services field, for example, funeral directors frequently must help grieving families and manage their clients' emotional distress, which may result in negative emotions. Funeral directors are expected to exhibit empathy, compassion, and support for the bereaved, even if it entails experiencing and expressing negative emotions [15]. However, some emotion regulation and emotional labor strategies could also fall under the category of emotion-focused coping, in contrast to problem-focused coping, [9]. For an extensive overview of the most prevalent coping theories, refer to Frydenberg [16] or Wong and Wong [17].

Recent methodological approaches acknowledge the momentary and dynamic nature of (within-person) emotion regulation processes [12]. To capture this dynamic nature, *ambulatory* or *ecological momentary assessment (EMA)* is the most suitable option. Since research on the antecedents and outcomes of emotional labor and emotional regulation in response to work-related demands using ecological momentary assessment is limited (i.e., EMA of emotional reactions to work-related demands and post-emotion regulation), we conducted a scoping review to identify research in this area. A scoping review differs from a systematic review in that it quickly outlines the essential concepts and types of evidence found in a research area, including the variables under investigation and relationships among them, without delving into the research findings in great detail. This type of review does not attempt to synthesize the evidence from the studies, nor does it assess the evidence's quality or determine whether the studies have produced robust or generalizable outcomes. Researchers can employ this tool to pinpoint deficiencies in current literature and gauge the feasibility (existence of valuable literature) and relevance (previous systematic reviews conducted) of conducting a complete systematic review [18].

### Theories of emotional labor and emotion regulation

Prominent theories that describing emotional labor include those developed by Hochschild [19] and Grandey and colleagues [20–22].

Customer service workers and employees are expected to adhere to integrative display rules, expressing positive emotions and hiding negative ones [22], with the purpose of enhancing the effectiveness of their interactions with customers by influencing them to purchase products, remain loyal to the organization, or spread positive word-of-mouth [19]. This may result in emotional labor since personal feelings may not always align with these emotional demands [21, 22]. One could imagine a customer service representative working for an online retail corporation who is handling a customer that received a damaged product due to a shipping mistake. The customer is understandably upset and frustrated and is communicating their anger and disappointment to the customer service representative. In this situation, customer service representatives must adhere to integrative display rules by maintaining a positive and empathetic tone, even though they may personally feel frustrated or even powerless to resolve the issue. They should conceal any negative emotions and respond in a reassuring manner that calms the customer. Hence, the customer service representative is required to engage in emotional labor by controlling their negative emotions and exhibiting a positive and empathetic demeanor. Although they may truly experience frustration due to shipping errors, it is vital for them to manage their emotions to enhance the effectiveness of the interaction and persuade the customer to remain loyal to the company by resolving concerns professionally and leaving a positive impression.

Two emotional labor strategies have been discussed: *Surface acting and deep acting* [19]. The former meaning deliberately concealing one’s true affective states or presenting false or inauthentic (e.g., amplified or downplayed) affective states and expressing emotions that are not genuinely felt using a broad range of outward emotional displays, including verbal cues, postures, and other nonverbal behaviors. The latter represents a deeper and more authentic method of emotional labor by modifying one’s inner feelings through techniques such as attention deployment, which entails shifting the focus of thoughts to things that induce the required emotions, or cognitive change, which involves evaluating or appraising situations differently to alter their emotional impact [20]. To illustrate the difference between these two strategies, one might envision two customer service representatives employed by the same company. Both receive a call from an angry customer who received a damaged product. Representative 1 feels irritated with the shipping department, having fielded similar complaints from customers previously. However, they have been trained to use surface acting. When speaking to the customer, they hide their feelings of frustration and annoyance and adopt a friendly and empathetic tone, assuring the customer that their issue will be resolved promptly. Internally, representative 1 may still feel upset, but on the surface, they present a calm and empathetic demeanor to prevent the situation from escalating. In contrast, representative 2 uses deep acting as their emotional labor strategy. When receiving the call from the upset customer, they genuinely empathize with the customer's frustration. They deploy attention to focus on the fact that the company should do better in terms of packaging and shipping. Representative 2 also engages in cognitive change by reframing the situation as an opportunity to improve the company's processes leading to genuine empathy and motivation to resolve the issue. The representative's language and tone towards the customer convey true empathy and a sincere intention to address the issue.

It is assumed that deep acting, as a strategy that modifies the felt emotion, is rather adaptive for organizational outcomes and for the mental health of employees, while surface acting, which only modifies the outward expression of the emotion, is seen as maladaptive [22–24]. However, meta-analytical findings demonstrate a weak relationship between both strategies and job performance [25, 26], and suggest that deep acting could have adverse effects on a person’s health [12]. It has been suggested that supplementing emotional labor findings with emotion regulation research could provide insight into the complex relationship between emotional labor and organizational and health outcomes based on these heterogeneous results. Grandey’s model utilizes emotion regulation as a guiding theory to understand the mechanisms of emotional labor that may lead to *stress* for individuals but bring benefits to the organization [20]. “Emotion regulation consists of the extrinsic and intrinsic processes responsible for monitoring, evaluating, and modifying emotional reactions, especially their intensive and temporal features, to accomplish one's goals.” [27]. Grandey used Gross' [28] process theory of emotion regulation, which distinguishes between antecedent- and response-focused emotion regulation. Antecedent-focused strategies are aimed at modifying affective states before they fully develop, while response-focused strategies are aimed at manipulating affective states after they have emerged [28]. Grandey [20] linked these two forms of emotion regulation to the work of Hochschild [19], mapping surface acting onto the response-focused strategies, whereby no attempt is made to change one's actual feelings, and deep acting onto the antecedent-focused strategies, whereby both behavior and internal experience are brought into alignment with organizational expectations [19, 20, 28]. Unlike emotion regulation, emotional labor *always* occurs with consideration for others, whereas emotion regulation can occur without assuming an interpersonal goal [8].

Both forms of emotional labor are self-regulation strategies that demand impulse control, suppression, and focused attention [28]. These demands are considered challenging for individuals due to their physiological and cognitive costs [19, 20, 28, 29]. More precisely, surface acting requires the suppression and inhibition of emotions, which can cause an unpleasant sense of inauthenticity [20] or *emotional dissonance,* defined as a sense of tension that occurs when experienced and displayed affect differ [19, 21, 25]. Deep acting, on the other hand, could alleviate emotional dissonance by aligning internal experiences with organizational expectacions in the short term [21], but in the long term it might have the harmful effect of causing a sense of alienation from one’s own feelings [19]. Both surface and deep acting may finally affect *job satisfaction *negatively, leading to *emotional exhaustion*, a facet of burnout, and *work withdrawal* [20, 22]. On the other side, Grandey [21] argued that job dissatisfaction may also trigger emotional labor as it impedes authentic displays of positive emotions and requires emotional labor.

Additionally, Grandey [20] introduced individual and organizational factors that may influence the process of emotion regulation at work and its outcomes. With regard to individual factors, she suggested that *gender* may influence the proposed relationships, with women being more experienced and possibly more skilled in emotional labor, but also more likely to regulate their emotions and perform more emotional labor. Furthermore, the model proposes that individuals with higher positive expressivity maybe better equipped to follow display rules, resulting in a reduced need for emotional labor. Other factors that influence emotional labor processes include e*motional intelligence* (EI), *self-monitoring* (SM), and *affectivity*. Brotheridge and Grandey [22] further added *felt challenge* as a moderator, whereby individuals who perceive their ongoing interactions as challenging steer their emotional experiences toward the positive leading to a sense of accomplishment.

Regarding organizational factors, Grandey [20] saw the organization’s *interaction expectancies* as a precursor of emotional labor, differentiating between the interactions’ frequency, duration and variety, and the display rules that can be overtly or subliminally framed. If certain emotions are expected by the organization, individuals may be compelled to engage in more emotional labor to adhere to these expectations. The second antecedent she proposes are positive or negative *emotional events*. An emotional event such as interacting with a difficult customer or receiving praise from coworkers and supervisors, may provoke more emotional labor when resulting in emotions that are discrepant from the organization’s display rules. Other organizational factors were introduced as possible moderators of emotional labor. Greater *job autonomy* was hypothesized to reduce the unpleasant feelings of emotional labor as a lack of control over events results in the experience of stress, emotional exhaustion and emotional dissonance [19, 20, 30], and *social support* from supervisors and coworkers may lessen emotional labor itself by fostering positive work environments that produce feelings and expressions of positive emotions, which most organizations expect [20].

### Social interaction model

Another viewpoint on emotional labor is that of the social interaction model [31–33]. Within a work context, service representatives enhance positive emotions during customer service to facilitate service interactions by promoting positive social responses from clients [32, 33]. Consistent with Grandey's theory [20], the social interaction model assumes that emotional labor benefits organizations by improving interactions with the customers but comes at a cost to employees [31]. Surface acting is believed to result in more negative customer reactions than deep acting because it is considered inauthentic [21, 31, 32]. This negative customer feedback on surface acting leads to *strain* for the employee [31]. Deep acting, on the other hand, is associated with positive social feedback due to its more genuine nature [21, 31, 32]. These negative and positive reactions of customers to emotional labor may consume or replenish social *regulatory resources*, with surface acting worsening and deep acting improving social reactions [31].

### Ego depletion theory

The ego depletion theory [34], predecessor of the strength model of self-control [29, 35, 36] and the strength model of self-regulation [37, 38], also empathizes these regulatory resources. The ego depletion model proposes that emotional labor, like any other demanding regulation, leads directly to the *exhaustion* of limited resources, which are needed to continue behavior regulation, and that *fatigue* serves as one indication of these resources diminishing [21, 34, 36]. Surface acting and deep acting both are effortful and intentional strategies, leading to the consumption of regulatory resources and impairment of subsequent self-control tasks in the workplace, but these effects may also spill over into home domains [39]. Self-control is “the capacity to override or alter one’s predominant (pre-potent, automatic) response tendencies. Akin to the colloquial notion of willpower.” [40]. Since surface acting requires higher levels of self-control than deep acting due to the need of continuous self-monitoring and suppression or faking of emotions, performing more on a given day should result in a more depleted self-control capacity at the end of that day [36]. This reduced self-control capacity decreases *job satisfaction* [21] and may, for instance, find expression in more *alcohol consumption*, as self-control is typically needed to avoid or limit alcohol intake [41]. Depleted self-control capacity could also result in *work withdrawal* as a form of rest. The affected individual requires rest to replenish drained resources and to return to the self-regulatory demands of the organization with renewed vigor [29].

### The basis of the current scoping review

In 2017, Grandey’s model of emotional labor as emotion regulation has been updated [8]. Antecedent- and response-focused strategies [28] are referred to as broad categories of emotion regulation strategies, the former including, e.g., deep acting, reappraisal and situation modification, and the latter including, e.g., surface acting and physiological modification. Furthermore, the model examines the influence of work environment, personal characteristics and situational factors of a higher order, moderating the effects of events at work on the emotional labor process and its outcomes.

Besides the growing interest in the impact of emotional labor and emotional experiences on occupational outcomes and mental health in the occupational literature [1], advances have been made in research methods. Emotional labor and emotion regulation processes were originally studied at the person level, highlighting variations in strategies employed by individuals (see, for instance, [25, 42]). Recent methodological approaches argue that *ambulatory* or *ecological momentary assessment (EMA)* is more appropriate for capturing this dynamic nature of (within-person) emotion regulation processes [12]. EMA “involves repeated sampling of subjects’ current behaviors and experiences in real time, in subjects’ natural environments. EMA aims to minimize recall bias, maximize ecological validity, and allow the study of microprocesses that influence behavior in real-world contexts.” [43]. This method is highly valued in behavioral sciences due to research indicating that memory is not an exact representation of reality. Instead, memory is rather constructed and distorted, influenced by personality traits and the characteristics of the experience itself, as well as by a person's current feelings [44–50]. Especially routine experiences and behaviors are difficult to remember because they lack uniqueness and salience compared to more infrequent experiences [43]. Since emotional labor is primarily performed under such routine situations, studies of this behavior are susceptible the memory processes described above and might therefore lack reliability in accurately reconstructing reality.

This scoping review uses Grandey and Melloy’s model [8] to organize the literature on different regulation strategies, influencing factors, and outcomes at the work context, person, and event level (i.e., the situational factors). The review focuses on changing emotional experiences and expressions through cognitive and emotional changes that comply with work-related (emotional) display rules. This does not only imply showing positive and hiding negative emotions, but also exhibiting negative and neutral emotional displays as well as following rules to convey irritation [8].

 Meeting the requirements of this antecedent- versus response-focused approach requires research methods that delve into these highly detailed temporal processes, which cross-sectional and longitudinal studies alone are inadequate to explore. To minimize bias in autobiographical memory retrieval and capture the emotional labor process without it distorting forthcoming memories, ecological momentary assessment is more preferable than cross-sectional and longitudinal methods [43]. Since research on the antecedents and outcomes of emotional labor using ecological momentary assessment is still scarce, a scoping review was conducted to identify research in this area. To guide the scoping review, we posed the following research question: What EMA based research activities do exist in the area of emotional labor as emotion regulation in the context of the workplace?

## Methods

The review article was prepared based on Arksey and O'Malley's five key stages [18]: (1.) identifying the research question, (2.) identifying relevant studies, (3.) study selection, (4.) charting the data, and (5.) collating, summarizing, and reporting the results. In addition, we were guided by the PRISMA checklist on scoping reviews [51]. No review protocol was preregistered.

**Fig. 1:**
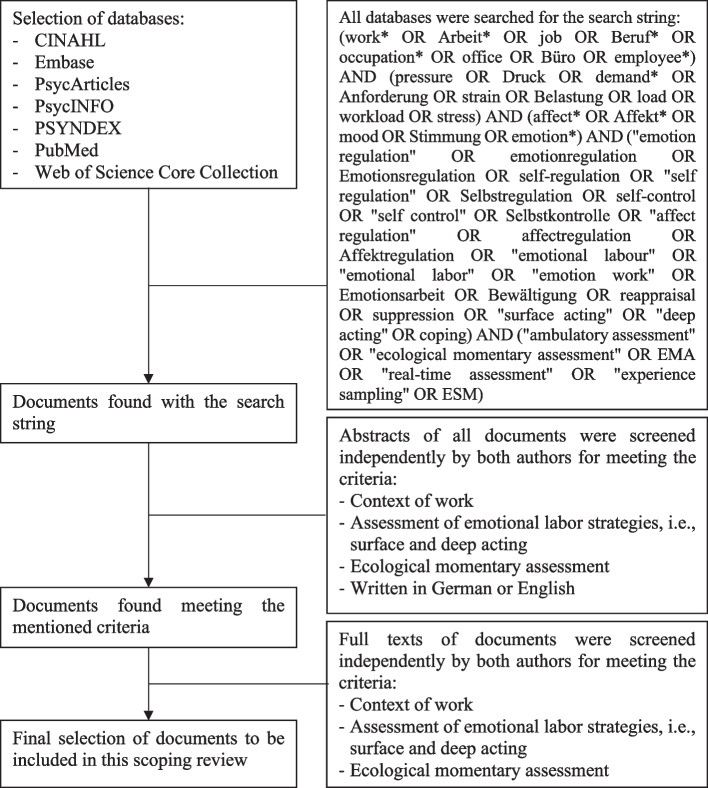
Final search and selection strategy

After formulating the research question ((1.) What EMA based research activities do exist in the area of emotional labor as emotion regulation in the context of the workplace?), we decided on the following inclusion criteria for this scoping review (2.) Papers needed to measure or focus on emotional labor strategies as outlined by Grandey and Melloy [8], i.e., surface acting and deep acting, as well as antecedents (e.g., job characteristics) or outcomes (e.g., mental health or job performance) connected with these strategies. To prevent omitting articles that examined these strategies but used related terms (e.g., reappraisal, suppression of emotional experience of Gross’ model), we expanded our search to comparable emotion regulation terms. Furthermore, the method of EMA had to be used to make sure emotional labor was captured as a *within-person process* instead of modeling strategies as relatively stiff tendencies that differ between persons. Regarding the population, there were no restrictions set as our objective was to provide a comprehensive overview of the research on emotional labor as emotion regulation, independent of country, culture, or sector. All papers were required to assess emotional labor or emotion regulation in the workplace with no restrictions as to time of day, workshift, day of the week, or year. The papers needed to be written in either German or English. To identify potentially relevant studies, searches were conducted in several databases without any time period restrictions. These databases included: Embase, PubMed, and Web of Science, as well as CINAHL, PsycArticles, PsycINFO, and PSYNDEX via EBSCO. This strategy was jointly developed by both authors, and the final search and selection procedure can be found in Fig. 1.

The first version of this scoping review has been created as part of a bachelor's thesis and encompasses publications up until the 14th of April, 2020. A subsequent search was conducted to extend the scope of our analysis. To select suitable studies (3.), both authors first screened all abstracts. In case of disagreements regarding their inclusion, the publications in question were discussed and a joint decision was made. Second, the full texts of the selected publications were thoroughly reviewed by both authors and a joint decision was reached in cases of disagreement. It should be noted that the full texts already included in the bachelor thesis, which lays the foundation for this extended research, were not reevaluated by the second author due to regulations requiring students to independently prepare their theses. These eight publications are not part of the subsequent analysis of interrater reliability. To evaluate interrater reliability for both abstract and full-text screening, Cohen's kappa statistic was used [52]. This statistical method is essential for ensuring data accuracy by quantifying the consistency of assessments between data collectors or raters. Jacob Cohen introduced Cohen's kappa in 1960 to address the limitations of the traditional percent agreement measure, which could not account for chance agreement. Cohen's kappa, a correlation statistic ranging from -1 to + 1, provides a more robust measure of interrater reliability by considering the possibility of raters guessing due to uncertainty [53].

Data were recorded on the publications' metadata, including information such as the authors, year, title, journal, abstract, and source. The studies’ theoretical background, hypotheses, EMA implementation, variables measured, methods, and results were also collected (4.). The authors collaboratively made the decision on the information and data extraction using forms in Excel and Word. In the last step (5.), the theoretical background and derived hypotheses were analyzed to illustrate the theories and previous findings upon which the research builds on and to determine whether the hypothesized relationships resemble or differ from each other. The studies’ hypotheses were extracted and organized according to their content to facilitate a comparison of the hypothesized relationships. As emotion regulation research primarily occurs in laboratory settings rather than in real-life contexts, the review examined the implementation of EMA in employees' work environments while also considering relevant variables. Data were collected related to study design, number of participants and observations, and assessment duration to assess study quality. The evaluation of the studies’ quality followed the recommendations of Balderjahn et al. [54] and Maas and Hox [55], which mandate a minimum of fifty units at level two (here: participants) for analyzing variance levels and interactions in two-level models. It has also been suggested to include at least 30 units at level one (here: single assessments) [56]. Finally, the findings of the included studies were charted and linked to Grandey and Melloy’s model of emotional labor as emotion regulation [8]. This review’s results will be presented in written form, along with tables and figures, and more detailed itemization and charts in the supplement [see Supplement].

## Results

### Selection of sources of evidence

**Fig. 2:**
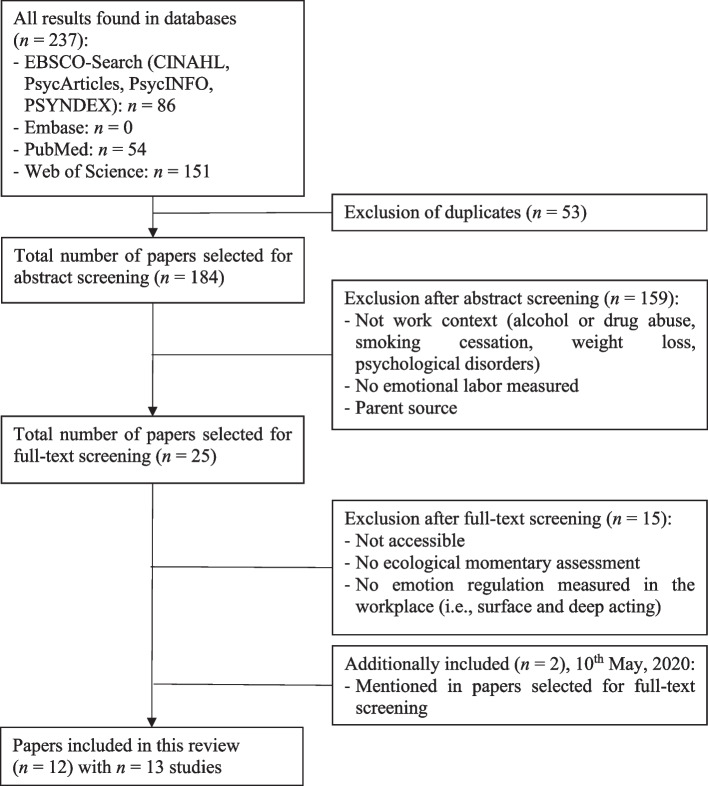
Flow diagram of selected studies

The scoping review was conducted initially as part of a bachelor thesis, encompassing publications until April 14, 2020, and a second search was conducted on February 2, 2022. The results of the two searches were combined and treated as one data set. After exclusion of duplicates, a total of 184 citations were identified through electronic database searches. Based on title and abstract screening, 159 publications were excluded due to not measuring emotion regulation in the workplace (e.g., focused on alcohol or drug abuse, smoking cessation, weight loss, or psychological disorders), not addressing emotion regulation strategies or because of a double registry of one article, which has been excluded manually. Of the remaining 25 articles selected for full-text screening, 15 were excluded for not adhering to EMA procedures, not measuring emotion regulation at the workplace in terms of deep acting and surface acting, or not being accessible. Despite contacting authors for access, these could not be obtained. Over the course of reading through the sources, two papers were further included, yielding a total of 12 publications with 13 studies included in this review (see Fig. 2). Cohen's kappa statistic was used to assess interrater reliability for both abstract and full-text screening [52]. It is pertinent to note that the calculation of Cohen's kappa was performed for the chosen papers prior to engaging in a thorough discussion and mutually agreeing on which papers to include. This involved an initial individual assessment of the papers by both authors, followed by collaborative discussions to resolve any differing opinions. Additionally, it is important to note that the analysis of interrater reliability for full-text assessment was limited to a subset of the selected studies. This limitation was due to the fact that the full-text analyses were conducted as part of a bachelor's thesis in 2020, which serves the foundation for this extended research. Thus, eight previously incorporated papers underwent no further evaluation by the second author. The interrater agreement was strong for the abstract screening (κ = 0.65) and moderate for the full-text screening (κ = 0.35).

The characteristics of the included studies will be presented according to the aforementioned criteria of theoretical background and hypotheses, EMA implementation and measured variables, and the studies’ results regarding the emotional labor strategies.

### Theoretical background and hypotheses of the studies

**Fig. 3:**
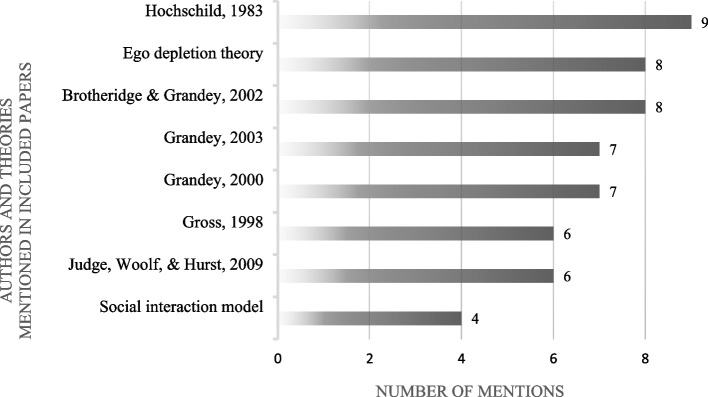
Relevant authors and theories in EL research (mentioned by at least four papers as theoretical background)

To ensure the clarity of a scoping review, only authors and theories deemed relevant - mentioned by at least four of the 12 included articles - will be discussed. This results in eight influential articles or theories that seem to be integral to emotional labor research (see Fig. 3). All of the 12 included articles built on an emotional labor theory as outlined in the following paragraphs, with Hochschild [19] being most frequently cited, followed by the works about emotional labor of Grandey and colleagues [20–22]. Adittionaly, half of the articles cited the work about emotion regulation by Gross's research on emotion regulation [28]. The social interaction model [31–33] was mentioned by four articles, the interrelated group of ego depletion theory [34], strength model of self-control [29, 35, 36] and strength model of self-regulation [37, 38] was cited by eight of the included papers. Six of the 12 articles also cited the work of Judge and colleagues [57]. This work is incorporated in the current scoping review and will be elaborated upon in the following sections. For a more detailed overview of the postulated links between emotional labor, its antecedents and outcomes in the individual studies, see Supplementary Tables S1 and S2 [see Supplement].

### EMA implementation

Nine of the thirteen studies applied an event sampling approach. This entailed gathering data on the start or end of the workshift or day, or participants ompleting a survey at a set time during the day. Three studies utilized a hybrid approach that included both time and event sampling. These studies randomly selected a designated event, such as a class period or a specific time during the day, for the assessment. In five of the studies, participants were given personal digital assistants (PDAs), i.e., small hand-held computers, to complete the surveys. Five studies made use of online surveys, three studies used paper- pencil questionnaires, and in one study a computer terminal was set up at the company’s headquarter. One study did not mention how EMA was implemented.

The analyses included 70 to 2051 single assessments, with 18 to 572 participants per study. Ten studies met the minimum of fifty units (here: participants) at level two, as suggested by Balderjahn et al. [54] and Maas and Hox [55]. All studies met the requirement of at least 30 units on level one (here: single assessments) suggested by Kreft and de Leeuw [56]. The drop-out rates ranged from three to 80 percent. Regarding personal assessments, participants could complete one to four assessments per day, according to the available data. Approximately half of the studies mentioned response rates, ranging from 12 to 90 percent.

The entire EMA period lasted between seven and 15 (work-) days, with six of the studies assessing data during ten workdays, two studies collecting data for seven days (possibly) including the weekends, and another four studies collecting data for 14 to 15 days. One study did not specify the duration of the data collection, only mentioning that the study lasted two weeks. For further details see Table 1.

**Table 1:** Characteristics of the assessment methods and observations of the included studies

	**Design**		**n Participants**			**Duration**	**Observations**					
**Study**	Sampling design	EMA device	Invited	Included in analysis	Drop-out rate	Possible time period (p.p.)	Possible observations (total)	Observations included in analysis (total)	Response rate (total)	Possible observations (p.p.)	Average of observations (p.p.)	Possible observations per day (p.p.)
Beal et al. (2013)	At the beginning of the shift, before the rush, after the rush and at the end of the shift (i.e., event-based)	PDA	84	63	25%^a^	15 workdays	3780^a^	2051	54%^a^	60^a^		4
Bono et al. (2007)	Within the shift (i.e., event-based) during each 2-h segment of the 8-h workday (i.e., random-based) including a 10-min response window	PDA	73	54	26%^a^	10 workdays	2266	889	39%	40^a^	15	4
Chang et al. (2021)	Three daily alerts at beginning of the day, mid-day planning period or lunch time, and end of the day (i.e., event-based). Optional additional assessment during the day following student misbehavior (i.e., event-based)	PDA (iPad Mini)	68	42 (pre-survey) & 15 (final EMA)	38% (pre-survey) & 78%^a^ (final EMA)	Two weeks	450^a^ & 600^a^ (without and with optional daily assessment, resp.)	70	16%^a^ & 12%^a^ (without and with optional daily assessment, resp.)	30^a^ & 40^a^ (without and with optional daily assessment, resp.)		3 & 4 (without and with optional daily assessment, resp.)
Huang et al. (2015)	In the middle and at the end of the workday (i.e., event-based)	PPQ	100	84	16%^a^	15 workdays	2520^a^	1054	42%^a^	30	25	2
Huppertz et al. (2020)	3 daily surveys sent out at 13:00, 16:00, and 19:00 with instructions to complete the survey during work, shortly after work, or in the evening, resp. (i.e., event-based)	Online survey	150	144 & 146 (depending on analysis)	4%^a^ & 3%^a^ (depending on analysis)	7 days	3024^a^ & 3066^a^ (depending on analysis)	699	23%^a^	21^a^		3
Judge et al. (2009)	At the end of the workday (i.e., event-based)	Online survey	230	127	45%	7 days, weekends included	635	398	63%	7	4.5	1
Keller et al. (2014)	At the beginning of each regular teaching lesson including a 5-min response window (i.e., event-based) and once within that lesson including a 5-min response window (i.e., random-based)	PDA (Palm Pilot Z22)		39		10 workdays	882^a^	794	90%		40	
Sayre et al. (2019, Study 1)	Participants were e-mailed a link twice a day for 14 days between 13:00 and 17:00 & between 17:00 and 21:00 (i.e., event- and random- based)	Online survey	219	86	61%^a^	14 days, weekends included	2408^a^	390	16%^a^	28^a^		2
Sayre et al. (2019, Study 2)	A survey link was sent electronically twice a day for 10 workdays at 9:00 & 17:00 (event-based)	Online survey	95	90	5%^a^	10 workdays	1800^a^	700	39%	20^a^		2
Scott et al. (2011)	Right before beginning the shift and before leaving work (i.e., event-based)	Online survey	68	58	15%^a^	10 workdays	580	405	73%	20^a^	14	2
Totterdell et al. (2003)	At 11:00, 13:00, 15:00, and 17:00 including a 30-min response window (i.e., event-based) with a 30-min response window	PDA	90	18	80%^a^	10 workdays	720^a^	537	85%	40^a^	29.83	4
Wagner et al. (2014)	Prior to beginning their shifts and following their shift, participants used a computer terminal to fill out two surveys, and each evening they completed a paper-based survey just prior to retiring to bed (i.e., event-based)	Online at a compu-ter termi-nal & PPQ	100	78	22%^a^	14 workdays	3276^a^			42^a^		3
Xiao et al. (2019)		PPQ	1000	572	43%^a^	10 workdays						

Blank spaces indicate that the information was not reported in the studies. *PDA* Personal d igital a ssistant. *PPQ* Paper- pencil questionnaire

^a^Values were calculated by this review’s authors

 All samples comprised service workers, such as salesclerks, restaurant servers, teachers, and bus drivers from the US, Canada, China, Taiwan, and Germany. The average age ranged from 23 to 52 years and the majority of the participants were female, with only two studies including more men than women. The average job tenure spanned from 1.8 to 16.16 years, with four studies not reporting job tenure information. Seven of the studies offered payment for participation, while the remaining studies did not mention any compensation. For additional details on the studies’ samples see Table 2 and Supplementary Table S3 [see Supplement].

**Table 2:** Sample characteristics of the included studies’ participants

Paper	Age (in years)	Gender (female)	Location	Ethnicity	Occupation	Job tenure (in years)	Payment
Beal et al. (2013)	*M* = 31.23, *SD* = 9.63	66%	Midwest USA, Canada		Restaurant servers from seven different restaurants	2.46	$50
Bono et al. (2007)	*M* = 41, *SD* = 10	94%		Caucasian (86%), African American (9%), Asian (5%)	Health care workers from family practice clinics (nurse, medical assistant, and lab technician, *n* = 14), administrative offices (patient services support, case analyst, and account follow-up specialist, *n* = 10), and a billing office (accountant, case manager, and human resource specialist, *n* = 33)	5.24	$25
Chang et al. (2021)	30.2% 31–40, 53.7% over 41 (pre-survey)	79%^1^ (pre-survey) & 80%^1^ (final EMA)	Southeast USA		Teachers from a Title-I middle school	*M* = 13, *SD *= 7.17 (pre-survey) & *M* = 14.41^1^, *SD* = 5.53^1^ (final EMA)	
Huang et al. (2015)	*M* = 23	73%	Midwest China		Employees working in the call center of a telecommunication company	1.8	Max. $30
Hupperts et al. (2020)	*M* = 31, *SD* = 10.2	52%			Employees from customer-facing jobs working with customers, defined as persons that buy a good or a service (51%) or as persons that receive help or advice from a professional person (23%), employees in hospitality working with guests (*n* = 5), with students (10%), patients (3%), children (3%), or none of the groups indicated above (4%); participants’ job titles indicated that most of them were working in sales (29%), IT (13%) and hospitality and related fields (10%)	*M* = 5.4, *SD* = 6.8	£0.35 for completion of a midday survey, £0.25 for an end-of-work survey, and £0.25 for a bedtime survey; bonus payment for completion of all three daily surveys (10%, 15%, 20%, 25%, and 30% for 1, 2, 3, 4, and 5 complete days, resp.); additional bonus payment of £1.00 for completing all daily surveys for five days
Judge et al. (2009)	*M* = 30.2, *SD* = 9.5	56%	25 different states of the USA	Caucasian (63.0%), Asian (22.8%), Hispanic (7.9%), African American (4.7%), other (1.6%)	Customer service workers	3.7	$50
Keller et al. (2014)	*M* = 44.14, *SD* = 11.33	56%	Germany		Secondary teachers	16.16	
Sayre et al. (2019, Study 1)	*M* = 35.68, *SD* = 10.5	63%	USA	White (83.7%), Hispanic (10.5%), Asian (2.3%), Black (4.7%), Native American (1.2%)	Employees with daily contact with individuals outside their organization (e.g., customers, patients, students, clients): professional roles (31.4%; financial services, managers, lawyers), caring work (18.6%; health care), other service roles (15.1%, beauty/salon, housekeeping), education (14.0%), sales (12.8%), food service (5.8%), hotel/hospitality (2.3%)	6.15	
Sayre et al. (2019, Study 2)	*M* = 31.63, *SD* = 8.89	73%	Taiwan		Nonstudents with traditional business hours (9:00-17:00) in jobs that involved daily contact with individuals outside their organization (e.g., customers, patients, students, clients); in food service (29.1%), professional services (21.5%, financial services, managerial, legal), sales (11.5%), health care (14.3%), education (6.3%), hotel and hospitality (4.7%), other services (12.6%, e.g., hair/beauty, housekeeping, etc.)	3.75	
Scott et al. (2011)	*M* = 48.4, *SD* = 8.6	37%	Northwest USA	African American (*n* = 16), Asian/ Pacific Islander (*n* = 2), Hispanic/ Latino (*n* = 1), white/Caucasian (*n* = 44); “other” category (*n* = 3), ethnicity not reported (*n* = 2)	Bus drivers working for the same transportation company		Random drawing for five $100 prizes
Totterdell et al. (2003)	*M* = 34.28, range 22–54	72%			Customer service employees from seven teams in one section of a call center of a major financial bank		
Wagner et al. (2014)	*M* = 52	15%	Northwest USA	Caucasian (61.5%), African American (23.1%), Hispanic/Latino (3.9%), Asian/ Pacific Islander (2.6%), American Indian or Alaskan Native (2.6%), “other” or not reporting ethnicity (6.8%)	Bus drivers of a transit company		Compensated for participation via random drawing of monetary awards
Xiao et al. (2019)	under 19 (11%), 20–29 (23%), 30–39 (34%), 40–49 (18%), over 50 (14%)	51%	China		Frontline employees (service providers) of 71 companies in the hospitality and catering industry; luxury hotel chains and restaurants in metropolitan areas; working in hotel reception and VIP housekeeping; administration, customer service, maintenance and food service		

Blank spaces indicate that the information was not reported in the studies

### Measured variables

#### Deep and surface acting as variables of interest

Next, we examine the variables measured according to Grandey and Melloy’s [8] framework of emotional labor as emotion regulation. Half of the studies included deep acting in their investigations, while all 13 studies measured surface acting (note that reappraisal and suppression of James Gross’ emotion regulation model [28] were defined as deep acting and surface acting, respectively). Furthermore, Huang et al. [58] measured participants’ felt challenge, which refers to “the positive appraisal of job demands that includes interpreting work requirements as potentials for rewards and opportunities for growth” (p. 1400). This definition resembles Gross' cognitive change in antecedent-focused emotion regulation strategies [28], which involves the “tendency to interpret events more positively than warranted” (p. 284). As this scoping review is based on the concept of emotional labor as a process, all included studies measured within-person changes of these emotional labor strategies.

#### Emotional labor antecedents

Emotional events form the basis of the model at the intraindividual or event level. Only Totterdell and Holman [59] analyzed the effects of emotional events concerning responses of customers and coworkers, which varied in levels of pleasantness over time. Huppertz et al. [60] defined within-person fluctuations in surface and deep acting as antecedent variables. When it comes to the aspect of felt emotions, two studies assessed discrete emotions experienced during the day, including anger, anxiety, and enjoyment [61]. One study investigated state anxiety as a mediator between emotional labor and emotional exhaustion [62].

The relationship between emotional events and emotional labor appears to be moderated by work role interaction expectations and work features at the work-context level, as well as individual traits at the person level [8]. Trait variables identified as antecedents in the reviewed studies include affect spin, emotional exhaustion, emotional expressivity, emotional intelligence (EI), and trait surface acting and deep acting (measured as habitual use of suppression and reappraisal) [59, 61, 63, 64]. Grandey and Melloy [8] suggest that emotional expressivity and EI moderate the relationship between the emotional event and the emotional labor process as well as between emotional labor and its outcomes. Totterdell and Holman [59] analyzed supervisor support and job autonomy as antecedents for emotional labor levels among employees on the work-context level. Most studies investigated direct associations with emotional labor instead of a moderation of the events-emotional labor association.

Regarding work role interaction expectancies, one study [63] investigated emotional job demands on a between-person level to measure the demands encountered by employees, which could be mapped onto the task aspect modeled by Grandey and Melloy [8]. This served as a moderator between emotional labor and its outcomes. By questioning whether the emotional events stem from customers or coworkers, Totterdell and Holman [59] introduced a relational aspect into their item, which moderates the effect emotional events on the emotional labor strategy. One study investigated service innovation, described as the “practice of creating value for customers, employees, and business owners through improved service and processes, innovation in the organizational system, technical characteristics, and industry capabilities.” (p. 2) [65]. This factor could translate into higher job demands owing to perpetually changing expectations from the organization and was therefore measured at the group level of the included organizations.

#### Emotional labor outcomes

In one study, deep and surface acting themselves were defined as outcomes [64]. Seven studies measured momentary event level effects of emotional labor, with five studies assessing daily emotional exhaustion, defined as a “state of depletion in which an individual is not able to fully exert him or herself psychologically or emotionally.” (p. 492) [62]. One study measured daily depletion, which can be described as a lack of willpower [66], while another study investigated momentary psychological effort [60] as a mediator between emotional labor and emotional exhaustion. Beal et al. [63] measured daily fatigue. Judge et al. [57] and Scott and Barnes [67] investigated the influence of emotional labor on state affect and another two studies investigated its influence on daily stress or strain [63, 68]. Huppertz et al. [60] measured the extent to which individuals experienced feelings of authenticity in a given situation. These variables could be mapped onto intrapsychic outcomes at the event level, as proposed by Grandey and Melloy’s framework [8]. Momentary interpersonal outcomes of emotional labor were examined by Totterdell and Holman [59] with the extend the participants managed to express designated emotions.

With respect to outcomes at the higher levels of Grandey and Melloy's framework [8], seven studies measured variables at the person level and four at the work-context level. Concerning employee wellbeing, Wagner et al. [62] investigated the impact of surface acting on daily night-time insomnia (health aspect) and strain-based work-to-family conflict (relation). Sayre et al. [66]) measured the effect of emotional labor on alcohol consumption (health). Belonging to the attitudinal aspect, daily job satisfaction was examined as being affected by both surface and deep acting [57, 58, 68]. The aforementioned effects on emotional exhaustion could also be subsumed under this aspect. Moreover, Xiao et al. [65] included employees’ mental health in their analysis, which could also be mapped onto the health aspect of employee wellbeing. At the work-context level, four outcomes were scrutinized, namely service performance (including performance, proactivity and expressed emotion), customer conflict handling, work withdrawal, and rewarding interactions with customers [58–60, 67].

#### Moderators and mediators

Grandey and Melloy [8] predicted that contextual factors at both the individual and workplace level would influence the relationship between emotional labor and its outcomes. With respect to the person level, one study assessed the moderating effect of trait affect spin on the daily effect of emotional labor on fatigue and strain [63]. The study investigated the moderating effects of gender on the relationships between emotional labor, daily work withdrawal, and state affect [67], as well as the moderating effect of extraversion on the state affect – emotional labor and daily job satisfaction – emotional labor relationship [57]. On the work-context level, emotional job demands were measured as interindividual variables moderating the effect of emotional labor on alcohol consumption [66]. Furthermore, Bono et al. [68] gathered information on the transformational leadership of employees' supervisors, which can be mapped onto Grandey and Melloy’s [8] aspect of social climate and managerial practices. Xiao et al. [65] also investigated how group-level positive and negative emotional contagion moderates surface acting’s influence on daily mental health, which can serve as a buffer or amplifier, respectively.

Five studies investigated mediator variables between emotional labor and its outcomes, including the mediating effect of state affect on emotional labor and job satisfaction [57], work withdrawal [67] and strain [63]. Additionally, another study found mediation effects of psychological effort, felt authenticity, and rewarding interactions on emotional exhaustion resulting from emotional labor [60]. Furthermore, the motive to detach from work was measured as a state variable by one study, mediating the influence of emotional labor on daily alcohol consumption [66].

The precise details on the variables’ measures, including information on the specific items, scales, sources, and reliability figures, can be found in Supplementary Table S4 [see Supplement].

### Results of the studies

**Fig. 4:**
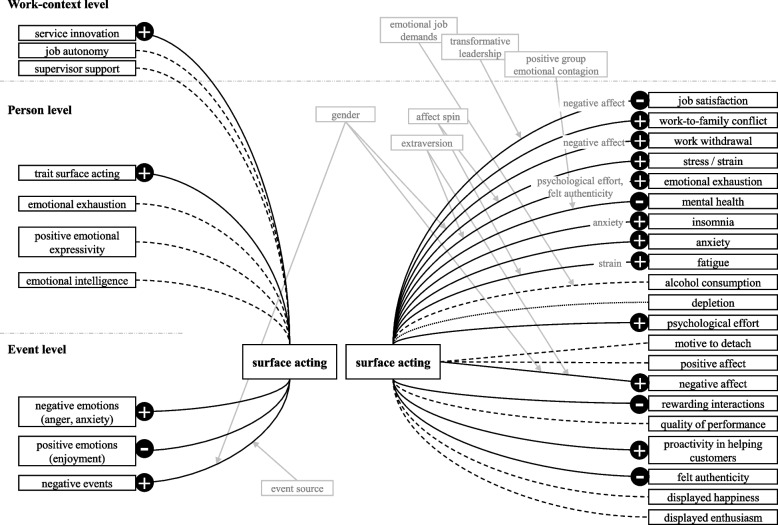
Results of the included studies with respect to surface acting. Note. The relationships between emotional labor and its corresponding antecedents and outcomes are displayed here, including only significant moderator (grey boxes with arrows indicating the moderated relationship) and mediator (grey terms overlayed on the respective relationship) effects. Surface acting has not been analyzed as a mediator. Dotted lines indicate no significant relationship was found. Drawn through lines indicate a significant relationship with a “+” or “-” implying a positive or negative effect, respectively. Dashed lines represent ambiguous findings

**Fig. 5:**
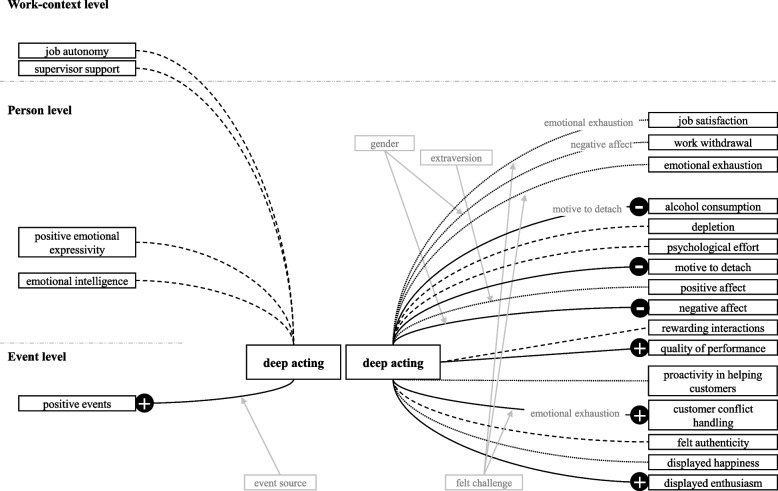
Results of the included studies with respect to deep acting. Note. The relationships between emotional labor and its corresponding antecedents and outcomes are displayed here, including only significant moderator (grey boxes with arrows indicating the moderated relationship) and mediator (grey terms overlayed on the respective relationship) effects. Deep acting has not been analyzed as a mediator. Dotted lines indicate no significant relationship was found. Drawn through lines indicate a significant relationship with a “+” or “-” implying a positive or negative effect, respectively. Dashed lines represent ambiguous findings

For an overview of the studies’ findings following Grandey and Melloy’s model of emotional labor as emotion regulation [8] see Figs. 4 and 5. Comprehensive presentations of the hypotheses and the measurement levels of the variables with traits at the person level and states at the event level, are available in Supplementary Tables S1 and S2. We interchangeably employ the terms "state" and "trait" with "event level" and "person level" as a significant portion of the reviewed studies do not adhere to the nomenclature proposed by Grandey and Melloy. It is important to note that the studies lack a clear delineation between work-context level and person level distinctions.

####  Associations between antecedent variables (at all levels) and event-level surface and deep acting

Positive events, rather than negative ones, were found to be correlated to deep acting, and only unpleasant events from customers, but not coworkers, were associated with surface acting [59]. There were no direct effects on emotional labor regarding job autonomy, supervisor support, or emotional expressivity, and emotional intelligence was only weakly related to emotional labor; specifically, it was only related to the deep acting strategy of positive refocus. One study discovered that trait surface acting is a significant predictor of state surface acting [61], while another found a significant relationship between service innovation and surface acting [65]. Keller et al. [61] found that anger (at the person level) was significantly related to surface acting, even after controlling for emotional exhaustion and trait surface acting. At the event level, anger and anxiety were positively related to surface acting, whereas enjoyment exhibited a negative relationship. Furthermore, upon controlling for trait surface acting, the correlation between emotional exhaustion and surface acting was close to zero. Chang and Taxer [64] discovered a significant dissimilarity in event-level emotional suppression (i.e. surface acting) between teachers with high trait deep acting (trait reappraisal) and low trait surface acting (trait suppression) and teachers that habitually use high deep acting and high surface acting.

####  Associations between event-level emotional labor and various outcomes, as well as moderators and mediators (at all levels)

##### Affect, affective experiences, stress, strain

State affect was investigated by two studies. The relationship between deep acting and negative affect was significantly negative [67], with females showing a stronger association than males. Additionally, Judge et al. [57] observed an overall negative influence of deep acting on positive affect being also negative. They also reported that deep acting was associated with lower levels of positive affect for introverts, but higher levels for extraverts. Scott and Barnes [67] found that deep acting was positively linked to positive affect with no moderating effect of gender. Surface acting positively predicted negative affect, with stronger relationships for extraverts than introverts [57] and stronger relationships for females than males [67]. There was no association found between surface acting and positive affect. Furthermore, gender had no influence on this relationship [67]. When it comes to concrete emotions, Wagner et al. [62] found that surface acting affects daily anxiety levels. Emotional exhaustion was influenced by surface acting [57, 58, 62, 69] but not deep acting [59], with the relationship between deep acting and emotional exhaustion being moderated by felt challenge [58]. The surface acting – emotional exhaustion relationship was stronger for females and partially mediated by negative affect [57], but not state anxiety [62]. Beal et al. [63] found a direct positive impact of surface acting on fatigue and an indirect effect on fatigue through psychological strain. In addition, they identified affect spin as a moderator of this effect, with high affect spin individuals displaying stronger relationships between surface acting and fatigue. Chang and Taxer [64] reported variations in experienced anger, emotional exhaustion, enjoyment, and feeling challenged among teachers who tend to engage in different patterns of emotional labor (low trait deep acting or high trait surface acting, respectively, vs. high trait deep acting).

Surface acting positively impacted stress and strain in employees, which was not moderated by the transformational leadership behaviors of the supervisors [68]. Beal et al. [63] found that the relationship between surface acting and strain was moderated by affect spin, with employees higher in affect spin suffering more from strain as a result of the positive effect of surface acting.

##### Mental health

Contentment, resilience, and peace of mind were negatively affected by surface acting, and this relationship was moderated by positive, but not negative group emotional contagion [65]. In their study, Wagner et al. [62] explored the effect of surface acting on night-time insomnia and found a positive association, which was partly mediated by state anxiety. Surface acting also increased work-to-family conflict, whereby the hypothesized mediating effect of state anxiety was not supported. Surface acting, but not deep acting, was marginally positively linked with emotional exhaustion [60].

##### Depletion, alcohol consumption

Huppertz et al. [60] report that event-level surface acting, but not deep acting, had a significant positive effect on psychological effort. In a separate study [66], researchers explored the relationship between deep acting and daily alcohol consumption and discovered a negative association between the two, even after accounting for negative affect and surface acting. They also hypothesized mediating effects of employees’ motive to detach from work and perceived regulatory depletion, but only the former was supported by their results. They did not find support for the hypothesized moderating effect of emotional job demands on the effect of deep acting on alcohol use. Regarding the outcomes of surface acting, they found no relationship between surface acting and alcohol consumption. Mediating effects of regulatory depletion were again not supported and the employees’ motive to detach from work was not found to mediate the relationship between surface acting and alcohol consumption. Emotional job demands moderated the effect; when demands were high, there was a positive effect of surface acting on alcohol consumption, and no effect when demands were low.

##### Job satisfaction, work withdrawal

Judge et al. [57] discovered that deep acting had no significant impact on job satisfaction, while Huang et al. [58] observed that job satisfaction was negatively affected by deep acting. Furthermore, this relationship was mediated by emotional exhaustion [58], but not positive affect [57]. The hypothesized moderating effect of extraversion on this relationship was not supported. Surface acting, on the other hand, affected job satisfaction negatively in both studies. State negative affect partially mediated this relationship, and there was no moderating effect of extraversion. One study [68] found a moderating effect of transformational leadership behaviors. When supervisors exhibited high levels of transformational leadership, the negative relationship between surface acting and job satisfaction diminished or vanished completely.

Scott and Barnes [67] examined the influence of emotional labor on work-withdrawal intentions. The study found no support for the hypothesis that deep acting has a negative effect on work withdrawal, but did find a positive relationship between surface acting and work withdrawal. This relationship was partially mediated by state negative affect, but not by positive affect. Additionally, the study found that gender had moderating effect on the direct effect of deep acting on work withdrawal, as well as on the indirect relationship through state negative affect, with both relationships being stronger for females. Gender had no significant influence on the indirect effect through positive affect. The above-mentioned positive surface acting – work withdrawal relationship was affected by the same moderating and mediating variables as the deep acting – work withdrawal relationship, albeit with slightly different effect magnitudes.

##### Customer conflict handling, rewarding interactions with costumers, authenticity, service performance

Huang et al. [58] found that deep acting had a positive impact on customer conflict handling. They also found that felt challenge moderates this effect, and that the positive impact is amplified with higher felt challenge. Adding emotional exhaustion to the equation, they found that this deep acting – felt challenge interaction, as well as the direct effect of deep acting, were both mediated by emotional exhaustion. Huppertz et al. [60] reported that momentary surface acting, but not deep acting, had a significantly negative impact on rewarding interactions and perceived authenticity. Totterdell and Holman [59] investigated service performance outcomes and found that deep acting was more strongly and positively associated with performance quality, displayed happiness, and proactivity in assisting customers, which all are facets of service performance, than surface acting. For further details on the findings of the studies see Supplementary Table S4 [see Supplement].

## Discussion

In this scoping review, we identified 12 studies addressing EMA-based research on emotional labor in terms of surface and deep acting published between 2003 and 2022.

### Theoretical background and hypotheses

In terms of theoretical background, this scoping review suggests widespread concurrence regarding the proposed relationships among emotional labor strategies, their antecedents, and outcomes. All studies in this field are rooted in emotional labor theories, with Hochschild’s theory [19] being the most commonly referenced, followed by that of Grandey and colleagues [20–22]. This is to be expected as these theories specifically aim at explaining the different relationships that exist within emotional labor, in contrast to the following theories, which derive emotional labor relationships from broader assumptions. Half of the studies utilized the emotion regulation theory by Gross [28], while four studies employed the social interaction model [31–33] and eight studies used the ego depletion theory [34] and related theories. In sum, research suggests that emotional labor is affected by job autonomy, supervisor support, and service innovation at the work-context level. Additionally, emotional expressivity, emotional intelligence, trait emotional labor and emotional exhaustion play a role at the person level, while affective events and emotions impact emotional labor at the event level, as proposed by Grandey and Melloy [8]. Emotional labor is hypothesized to impact various work-related outcomes such as work withdrawal, customer conflict handling, and service performance on the work-context level. In addition, it may impact the employees' mood (negative affect/positive affect), mental health, insomnia, job satisfaction, alcohol consumption, depletion, and conflicts with the family on the person level. Furthermore, emotional exhaustion, fatigue, stress, and strain may be experienced on the event level. It is evident that the studies included in this review solely focused on surface and deep acting because of this review’s focus. However, Grandey and Melloy [8] suggested incorporating more strategies such as physiological modification or attentional deployment. Furthermore, all 12 studies were conducted in environments reinforcing display rules that demand the expression of integrative emotions and the suppression of differentiating emotions. Differentiating and masking display rules were not taken into account. Certain job types require the expression of emotions such as fear or anger according to specific display rules (i.e., differentiating display rules; [70, 71]). Alternatively, other job types involve emotion control (i.e., masking display rules).

Future research should consider additional emotional labor strategies, whereby it is still unclear to what extent surface and deep acting vary from other emotion regulation techniques such as suppression and reappraisal [8, 42]. Both theoretical and empirical research should investigate the similarities and differences between emotional labor concepts. Additionally, the impact of differentiating and masking display rules on emotional labor must be investigated. Emotional job demands that require employees to show negative emotions may lead to other emotional labor outcomes than integrative demands, which require employees to show positive emotions. Such research should be conducted in work fields besides customer service areas. Researchers could examine the emotional labor of bill collectors or judges. According to the ego depletion theory [34], some outcomes hinge on the employees’ self-control resources, which are more exhausted in highly demanding emotional roles. Therefore, emotional job demands must be considered as a confounding variable in future studies. Otherwise, the results may be biased due to ground or ceiling effects.

### EMA implementation and measured variables

The studies included in the analysis measured emotional labor for one to three weeks encompassing a wide range of situations and increasing reliability compared to one-day measures. Nonetheless, future research is needed to determine whether the results of one-week studies resemble those of longer studies, which could indicate the presence of previously unconsidered variables. Between 18 and 572 employees used a PDA or an online survey to provide feedback on their use of emotional labor during certain times or marked events while at work or at home, resulting in 70 to 2051 individual assessments. Although a sample of only 18 participants [64] does not meet the requirements for multilevel analyses [54, 55], the overall sample sizes of the studies appear to be adequate, as further evaluated in the quality review below. Response rates varied widely, ranging from 12 to 90 percent. This should be viewed critically, as small response rates could indicate selection bias, potentially distorting study results. Future studies should aim to achieve a response rate of at least 50 percent, as demonstrated by Baruch and colleagues [72, 73]. Given that EMA occurs in real-life settings, there are several potential confounding variables that might distort the data. In the work context, this could be the employees’ salary or, as noted by Grandey and Melloy [8], the social climate at work. Future studies should be attentive to these potential confounders identified in this study while also considering the possibility of additional confounding variables. Furthermore, participants’ awareness of being observed may have led to reactivity and social desirability biases in the data. It is worth noting that social desirability may have been strengthened by receiving payment for participation in the studies, despite not being mentioned in the current scoping review’s results. It should also be noted that the included EMA data still relies on self-reports which are prone to potential inaccuracies such as lying or misunderstandings. Future studies may consider implementing more objective methods, such as measurement of heart rate or cortisol levels, as is frequently done in stress research [74–76]. While most of the reported studies used hierarchical level modeling to investigate between- and within-person variables, they did not specify quality standards for using multilevel modeling (e.g., [54–56]). Nevertheless, it may be presumed that these studies meet the quality requirements of the cited authors. Three studies did not meet the requirement of at least fifty units at level one [54, 55]. Future studies can use these studies as a guide for operationalizing quality through participant and observation numbers. All 13 studies assessed surface acting and half of them assessed deep acting. The majority of variables were measured daily within individuals. The more chronic outcomes suggested by Grandey and Melloy [8], such as alcohol consumption or job satisfaction, were also evaluated on a daily basis as state-like variables. Here, an inconsistency between theory and practice is evident in the research. To further test Grandey and Melloy’s model, researchers should measure the suggested chronic outcomes on the personal and work-context level using overarching or trait measures, rather than momentary measures. No study has yet measured the possible interactions between emotional labor strategies, e.g., faking happiness and suppressing anger simultaneously. These potential interactional effects should be addressed by future researchers. Moreover, certain constructs may significantly overlap and could be incorporated into a higher-level variable, e.g., fatigue, emotional exhaustion, and depletion. It remains also unclear, to what extent suppression and surface acting are either consistent with or differ from one another. It is crucial to disentangle these constructs for a more comprehensive understanding of emotional labor. Finally, none of the studies explored the trainability of emotional labor, which is why future research ought to investigate whether employees are able to improve their emotional labor use over the course of weeks or months. This implies longer or repetitive assessments of the same individuals.

### Results of the studies

Surface acting was influenced by negative events, trait surface acting and deep acting, service innovation and certain emotions, while deep acting was only influenced by positive events and emotional intelligence. All studies, except for one which did not formulate clear hypotheses, found effects consistent with the hypotheses. Specifically, positive events led to increased deep acting, contrary to the hypothezised decrease in deep acting. As stated by Grandey and Melloy [8] and supported by current research, emotional labor yields advantages for the organization, such as better customer conflict handling and service performance. However, it also results in drawbacks for the organization, as employees exhibit more work withdrawal behaviors. Conversely, for employees emotional labor caused more harm than benefits, resulting in emotional exhaustion, anxiety, negative affect, fatigue, and stress at the event level. Moreover, it led to job dissatisfaction, insomnia and family conflicts as more far-reaching consequences. These results supports the general notion that emotional labor may benefit the organization, but harms the employee [19, 20]. From a practical point of view, organizations should evaluate the impact of emotional job demands they pose on their employees. Dissatisfied, stressed and exhausted employees do a worse job than satisfied and rested ones [77–79], which might in turn negatively affect economic and financial figures. Therefore, it is crucial for organizations to address emotional job demands and promote employee well-being.

### Limitations of the scoping review

The scoping review at hand has certain limitations. To make this review more feasible, the review was limited to studies measuring surface and deep acting with ecological momentary assessment. As such, the results an only be assumed to have generalizability for the emotional labor strategies of surface and deep acting. Additionally, the results are only appliable to work environments with integrative display rules, since the included studies solely investigated service employees who had to show positive and conceal negative emotions. Furthermore, objective measures as heart rate or cortisol levels were not included in the search, but it can be assumed that this search would not have yielded significant results. Moreover, our search was restricted to EMA and synonyms thereof, neglecting terms as “diary” or “mobile”. A search string including these terms might have found more studies that could be mapped onto the method group of EMA, although the studies did not explicitly use this term themselves. However, we aimed to exclusively incorporate studies that adhere to the methodological recommendations for ecological momentary assessment, thus ensuring high-quality results. Other important studies might be missing due to not being included in the electronic databases searched. Future researchers might therefore benefit from searching in technical journals and conference transcripts for the most recent findings.

## Conclusion

The aim of this scoping review was to systematically map EMA-based research in the area of emotional labor as emotion regulation in the context of the workplace. The results are indicative of the new and emerging research domain with limited studies conducted so far. The findings of the studies align with Grandey and Melloy’s proposed relationships between antecedents and outcomes of surface and deep acting [8]. However, due to the limited number of studies, the findings should be replicated to increase the reliability of the results and allow for practical implications to be drawn. A thorough examination of Grandey and Melloy's model, encompassing the differentiation between work- context, personal, and event level, has not yet been conducted. At the event level, the model designates emotional labor as a mediator. In contrast, the majority of the studies within our review have predominantly explored emotional labor solely as either an outcome or an antecedent. Moreover, the precise allocation of predictors and outcomes to the event level (intra-individual, momentary assessment) and the personal level (inter-individual, state or trait assessment) has not been clearly defined. The lack of evidence for some hypotheses suggests the existence of unresolved relationships to be discovered. Nevertheless, Grandey and Melloy’s model of emotional labor as emotion regulation [8] serves as a useful guide for future research, providing several testable hypotheses. Future studies should follow the suggestions of the model by distinguishing between work-context, person, and event level, and incorporating EMA on the event level. This approach can help researches advance the understanding of emotional labor as emotion regulation. Systematic reviews are currently not advised due to the sparsity of evidence on the topic at hand. We are only at the beginning of EMA-based emotional labor research and are intrigued technologies and possibilities that the future holds.

### Supplementary Information

**Additional file 1: Tables S1-S4.**

## Data Availability

All charted results of the literature search are available in the manuscript, in the supplementary material and here: https://osf.io/6pa9r.

## Abbreviation

EMA: Ecological momentary assessment
