# Supplementary material for: Emotional labor as emotion regulation investigated with ecological momentary assessment – a scoping review
Source: BMC Psychol. 2024 Feb 12;12:69. doi: 10.1186/s40359-023-01469-9 (PMC10863272; doi:10.1186/s40359-023-01469-9)
Supplement: Supplementary file 1 — Additional file 1: Tables S1-S4. [file 40359_2023_1469_MOESM1_ESM.docx]

**Supplement**

**Table S1**

*Hypothesized antecedents of EL and their theoretical background*

| Antecedent | Hypothesized relationship | Theoretical background (shortened literal citations from the reviewed studies) | Examined by |
| --- | --- | --- | --- |
| Emotional Exhaustion | Emotional exhaustion (trait) is positively associated with SA (state). | The pertinent ER strategy in the context of teacher burnout and particularly regarding emotional exhaustion is SA (Chang, 2013; Näring et al., 2006). Emotional exhaustion, being an emotionally negative experience, is thought to provoke more ER in contexts where positive emotions are deemed appropriate. | Keller et al. (2014) |
| Emotional expressivity | Employees’ level of emotional expressivity (trait) will be negatively related to their levels of ER (state). | Grandey (2000) arguments that persons high in positive expressivity are more able to meet display rules and therefore require less ER. | Totterdell and Holman (2003) |
| Emotional intelligence | Employees’ level of emotional intelligence (trait) will be positively related to their levels of ER (state, (measured within-individuals). | ER is one of the components of emotional intelligence (Salovey & Mayer, 1990), so individuals with greater emotional intelligence might be more likely to use ER (Grandey, 2000). | Totterdell and Holman (2003) |
| Job autonomy | Job autonomy (trait) is positively related to employees’ levels of ER (state). | Grandey (2000) argued that greater autonomy (measured within-individuals) of the job role may affect the level and type of ER which employees engage in. A perceived lack of control over work events has been associated with unpleasant feelings (Hochschild, 1983), which may increase the level of ER needed to express positive emotions. | Totterdell and Holman (2003) |
| Negative emotions | Negative emotions (anger, anxiety; state) are positively associated with SA (state). | Negative emotions make it difficult to engage in authentic displays of positive emotions, leading to the need for one to deep or surface act (Grandey, 2000, 2003; Hochschild, 1983). | Keller et al. (2014) |
| Negative events | Negative events (state) from customers and coworkers are positively associated with EL (state). | Negative events lead to negative emotions that make it difficult to engage in authentic displays of positive emotion because they induce negative emotions, leading to the need for one to deep or surface act (Grandey, 2000, 2003; Hochschild, 1983). | Totterdell and Holman (2003) |
|  | The positive association between negative events (state) and EL (state) will be moderated by the event’s source (state), with the relationship being stronger for customer than coworker events. | Grandey (2000) proposed that affective events from customers would have a greater impact on ER than those from coworkers because display rules are more explicit for customer interactions. Furthermore, service agents’ amplified positive emotions during customer service to facilitate service interactions and foster positive social responses from customers but not coworkers (Côté & Morgan, 2002; Darley & Fazio, 1980). | Totterdell and Holman (2003) |
| Positive emotions | Positive emotions (enjoyment, state) are negatively related to SA (state). | Positive emotions make it easier to engage in authentic displays of positive emotion. Thus, there is less need for one to deep or surface act (Grandey, 2000, 2003; Hochschild, 1983). | Keller et al. (2014) |
| State SA, State DA | SA and DA (state) positively relate to psychological effort (state), with SA - psychological effort relationship being stronger.  There is a positive indirect relationship between SA and DA (state) and emotional exhaustion via psychological effort (state). | Psychological effort is an important mechanism proposed in the emotional labor literature to explain relationships of emotional labor strategies with well-being and strain, including emotional exhaustion (Beal et al., 2013; Côté, 2005; Grandey, 2003; Huang et al., 2015). Accordingly, engaging in surface and deep acting requires the expenditure of resources, which may lead to feelings of mental exhaustion and fatigue in the short run (Huang et al., 2015; Xanthopoulou et al., 2018), and to burnout in the long run (Hulsheger & Schewe, 2011). | Huppertz et al. (2020) |
| Supervisor support | Supervisor support (trait) will be positively related to employees’ levels of ER (state). | Support from supervisors may reduce ER by creating a positive work environment in which it is easier for employees to feel and express the positive emotions that are expected by their organization (Grandey, 2000). | Totterdell and Holman (2003) |
| Trait SA | Trait SA is positively associated with state SA. | Theoretical considerations (Robinson & Clore, 2002) and empirical investigations indicate that trait-reports on emotional experiences can be somewhat biased and do not necessarily reflect an individual’s actual – state-level – experiences, i.e. *trait-state discrepancy* (Keller et al., 2014). Also, studies employing trait-reports only address inter-individual differences in emotional experiences, and not much is known about how the experiences of emotions and ER are related on an intra-individual level. Furthermore, pertinent EL theories (Grandey, 2000) are based on intra-individual, that is, situation-specific considerations, such as in specific situation when employees experience an inappropriate emotion, they suppress that emotion, thereby draining their resources. That these relationships also extend to the inter-individual level, that is, between employees, is implicitly assumed, yet this may not necessarily be the case and needs some kind of testing. | Keller et al. (2014) |
| Trait SA (suppression), Trait DA (reappraisal) | reappraisal (trait) and suppression (trait) influence the momentary usage of response modulation strategies (state, SA: suppress emotions, and fake positive emotions) in response to student misbehavior (state).  reappraisal (trait) and suppression (trait) influence teachers’ affective experiences in the classroom (state). | A teacher may try to alter physiological, experiential, or behavioural aspects of their emotional response. This strategy is referred to as response modulation (Gross, 2015) and can be further broken down to suppression, faking (Grandey, 2000), and masking. Suppression has been found to be detrimental to teachers’ well-being (Chang, 2013), whereas cognitive reappraisal is generally considered an adaptive emotion regulation strategy. Lee et al. (2016) found that reappraisal is positively correlated with enjoyment. However, some researchers have found that reappraisal does not significantly mediate the relationship between teachers’ perceptions of student misbehaviour and emotional exhaustion (Chang, 2013). This could be because teachers are using different regulation strategies to regulate their emotions in response to student misbehaviour (Gross & Thompson, 2007). | Chang and Taxer (2021) |
| *Note*. EL = Emotional Labor. ER = Emotion Regulation. SA = SA. DA = DA. State = event-level, trait = person-level | | | |

**Table S2**

*Hypothesized outcomes of EL and their theoretical background*

| Outcome | Hypothesized relationships | Theoretical background | Examined by |
| --- | --- | --- | --- |
| Affective experiences | Thus, the aim of Study 2 was to examine if teachers’ habitual patterns of reappraisal and suppression, that is their tendency to reappraise or suppress irrespective of the situation, influence their momentary usage of response modulation strategies in response to student misbehaviour. We further examined how these habitual patterns of emotion regulation influence teachers’ affective experiences in the classroom. | A teacher may try to alter physiological, experiential, or behavioural aspects of their emotional response. This strategy is referred to as response modulation (Gross, 2015) and can be further broken down to suppression, faking (Grandey, 2000), and masking. Suppression has been found to be detrimental to teachers’ well-being (Chang, 2013), whereas cognitive reappraisal is generally considered an adaptive emotion regulation strategy. Lee et al. (2016) found that reappraisal is positively correlated with enjoyment. However, some researchers have found that reappraisal does not significantly mediate the relationship between teachers’ perceptions of student misbehaviour and emotional exhaustion (Chang, 2013). This could be because teachers are using different regulation strategies to regulate their emotions in response to student misbehaviour (Gross & Thompson, 2007). | Chang and Taxer (2021) |
| Alcohol consumption | DA (state) is negatively associated with alcohol consumption (state). | DA can result in resource gains such as positive social feedback (Côté, 2005; Côté & Morgan, 2002) and reduced exhaustion, which are negatively associated with alcohol consumption (Xanthopoulou et al., 2018). | Sayre et al. (2019) |
|  | The DA – alcohol consumption relationship is mediated by perceived regulatory depletion (state). | Côté (2005) posits that DA replenishes regulatory resources by improving social reactions or by reducing exhaustion (Xanthopoulou et al., 2018). | Sayre et al. (2019) |
|  | The DA – alcohol consumption relationship is moderated by emotional job demands (trait), with higher emotional job demands weakening the relationship. | Continuous effort of ER due to high emotional job demands is a stressor on employees that draws on their regulatory resources. Thus, more ER will draw on the already limited resource of self-control(Baumeister et al., 2007). | Sayre et al. (2019) |
|  | The conditional DA – emotional job demands effect on alcohol consumption is mediated by depletion (state). | DA is thought to replenish regulatory resources (Xanthopoulou et al., 2018), whereby high emotional job demands lead to regulatory depletion, which in turn decreases the self-control capacity to avoid or limit alcohol consumption (Baumeister et al., 2007). | Sayre et al. (2019) |
|  | The DA – alcohol consumption relationship is mediated by the motive to detach from work (state). | The process model of depletion proposes an alternative to the limited capacity explanation, instead arguing that temporary shifts in motivation and attention explain regulatory impairment from one task to another (Inzlicht & Schmeichel, 2012; Inzlicht et al., 2014). Given the benefits of DA e.g. positive social feedback (Côté, 2005; Côté & Morgan, 2002) and reduced exhaustion (Xanthopoulou et al., 2018), when employees deep act more than normal they are less motivated to turn attention away from the work role, instead they can continue to enjoy and exploit the rewards e.g., thinking about a friendly customer after work. | Sayre et al. (2019) |
|  | SA is positively associated with alcohol consumption (state). | Grandey et al. (2019) and Shepherd et al. (2019) found a link between SA and alcohol use. SA consumes regulatory resources that are needed to avoid or limit alcohol consumption (Baumeister et al., 2018; Muraven & Shmueli, 2006). | Sayre et al. (2019) |
|  | The SA – alcohol consumption is mediated by perceived regulatory depletion (state). | SA consumes regulatory resources that are needed to avoid or limit alcohol consumption. The reduced self-control capacity makes it more difficult to avoid or limit alcohol consumption (Baumeister et al., 2018; Muraven & Shmueli, 2006). | Sayre et al. (2019) |
| Alcohol consumption | The SA – alcohol consumption is moderated by emotional job demands (trait), with higher emotional job demands strengthening the relationship. | Continuous effort of ER due to high emotional job demands is a stressor on employees that draws on their regulatory resources. Thus, more ER will draw on the already limited resource of self-control (Baumeister et al., 2007; Muraven & Baumeister, 2000; Muraven et al., 1998). | Sayre et al. (2019) |
|  | The conditional SA – emotional job demands effect on alcohol consumption is mediated by depletion (state). | Both SA and high emotional job demands (Baumeister et al., 2018) lead to regulatory depletion, which in turn decreases the self-control capacity to avoid or limit alcohol consumption (Muraven & Shmueli, 2006). | Sayre et al. (2019) |
|  | The SA – alcohol consumption is mediated by the motive to detach from work (state). | The process model of depletion proposes an alternative to the limited capacity explanation, instead arguing that temporary shifts in motivation and attention explain regulatory impairment from one task to another (Inzlicht & Schmeichel, 2012; Inzlicht et al., 2014). SA requires the suppression and inhibition of emotion, resulting in the unpleasant sense of being inauthentic (Grandey, 2000; Hochschild, 1983). As such, SA on a given day is likely to motivate a shift away from the work role, distancing oneself from this unpleasant inauthenticity, and toward more rewarding or gratifying behavior (e.g., alcohol use). | Sayre et al. (2019) |
| Anxiety | SA (state) is positively associated to anxiety (state). | Viewing the self as coherent is extremely important and personally meaningful and an individual might threaten his or her sense of self by acting contrary to what he or she internally experiences at a given moment (Grandey, 2003), which can lead to anxiety (Lazarus & Folkman, 1984). | Wagner et al. (2014) |
| Customer Conflict Handling | DA (state) is positively associated with customer conflict handling (state). | The way in which employees manage their feelings and expressions can influence the effectiveness of their interactions with customers (Hochschild, 1983), with authentic positive display through DA appearing more sincere and thus eliciting more positive social feedback from customers (Côté, 2005; Darley & Fazio, 1980). Furthermore, if agents’ emotional resources are depleted in the short term, they are less likely to effectively regulate their emotions to handle potential customer conflict in their daily interactions (Baumeister et al., 1998; Baumeister & Vohs, 2016). | Huang et al. (2015) |
|  | The DA – customer conflict handling relationship is moderated by felt challenge (state), with higher felt challenge strengthening the relationship. | When feeling challenged, agents are more likely to see personal resources at their disposal as exceeding situational demands (Blascovich & Tomaka, 1996). When employees do not perceive their service interactions as challenging, they may construe their tasks as boring or taxing – the “default mode” in some service settings(Callaghan & Thompson, 2002). In such cases, the limited resource input to DA makes it less effective in generating affective, social, and person resources. As a result, resource consumption and gains are offsetting. Perceiving challenges enhances the DA process by representing an additional venue for personal growth and work satisfaction. | Huang et al. (2015) |
|  | The conditional DA – felt challenge relationship on customer conflict handling is mediated by emotional exhaustion (state). | The availability of emotional resources associated with fewer exhaustion will enable employees to better attend to customers’ needs and to solve potential conflicts which consume high levels of emotional and attentional resources(Palmatier et al., 2006). When emotionally exhausted, employees have limited resources to draw on and invest less of their attention in their immediate customer interaction (Lee & Ashforth, 1990, 1996; Rodell & Judge, 2009). | Huang et al. (2015) |
| Emotional exhaustion | EL (state) is positively associated with emotional exhaustion (state) in employees. | EL can have negative consequences for employees as, e.g., burnout (Hochschild, 1983). Particularly, Morris and Feldman (1996) found DA and SA relating to burnout, especially to the dimension of emotional exhaustion. | Huang et al. (2015) and Totterdell and Holman (2003) |
|  | SA ’s (state) relationship with emotional exhaustion (state) is stronger than the relationship between DA (state) and emotional exhaustion (state). | SA has been found to have stronger associations with emotional exhaustion than DA (Brotheridge & Grandey, 2002; Richards & Gross, 2006) and daily well-being is in general affected more by SA than DA (Grandey, 2000). | Totterdell and Holman (2003) |
|  | The DA – emotional exhaustion relationship is moderated by felt challenge, with lower felt challenge strengthening the relationship. | When feeling challenged, agents are more likely to see personal resources at their disposal as exceeding situational demands (Tomaka et al., 1993), resulting in some kind of emotional reserve in the form of motivational resources. When employees do not perceive their service interactions as challenging, they may construe their tasks as boring or mundane (Callaghan & Thompson, 2002). In such cases, the limited resource input to DA makes it less effective in generating affective, social, and person resources, leading to more exhaustion. | Huang et al. (2015) |
|  | SA (state) is positively associated with emotional exhaustion (state). | EL, especially SA, has generally negative consequences for employees as, e.g., burnout (Grandey, 2000; Hochschild, 1983). Brotheridge and Grandey (2002) found a significant relationship between SA (e.g., hiding anger and fear) and emotional exhaustion as it is effortful and consumes regulatory resources(Baumeister et al., 1998; Baumeister et al., 2018). | Judge et al. (2009) and Wagner et al. (2014) |
|  | There is a positive indirect relationship between SA and DA (state) and emotional exhaustion via psychological effort (state). | Engaging in surface and deep acting requires the expenditure of resources, which may lead to feelings of mental exhaustion and fatigue in the short run (Huang et al., 2015; Xanthopoulou et al., 2018), and to burnout in the long run (Hulsheger & Schewe, 2011). | Huppertz et al. (2020) |
|  | There is a positive indirect relationship between SA (state) and emotional exhaustion (state) via felt authenticity (state).  There is a negative indirect relationship between DA (state) and emotional exhaustion (state) via felt authenticity (state). | Surface acting is likely to result in the subjective experience of inauthenticity, which causes strain and has been associated with burnout (Grandey & Gabriel, 2015). In contrast, deep acting involves changing the underlying emotions so that the resulting emotions are aligned with the inner self (Brotheridge & Lee, 2002; Goldberg & Grandey, 2007). Deep acting involves the genuine display of required emotions, which in turn allows for the authentic expression of the self. | Huppertz et al. (2020) |
|  | There is a positive indirect relationship between SA and emotional exhaustion via rewarding interactions.  There is a negative indirect relationship between DA and emotional exhaustion via rewarding interactions. | Surface acting results in inauthentic emotional expressions. According to Côté’s (2005) social interaction model, this evokes negative reactions in the interaction partner and these negative reactions, for instance anger, disappointment, or disrespect, function as stressors that induce strain and affect the employee’s well-being. Accordingly, surface acting can be expected to lead to emotional exhaustion via reductions in rewarding interactions. | Huppertz et al. (2020) |
|  | The indirect positive relationship between DA and emotional exhaustion via psychological effort (Hypothesis 2b) and the indirect negative relationships between  DA and emotional exhaustion via felt authenticity (Hypothesis 4b) and via rewarding interactions (Hypothesis 6b) attenuate each other. |  | Huppertz et al. (2020) |
| Emotional exhaustion | The SA – emotional exhaustion relationship is moderated by extraversion (trait), with the relationship being stronger for introverts. | Individuals experience more positive subjective outcomes when they behave in a manner consistent with their trait (Little, 2000; Moskowitz & Coté, 1995). Bono and Vey (2007)argued that behaving in the positive manner required by most customer service jobs should be uplifting for extraverts, who tend toward positive emotion(Fleeson et al., 2002; McNiel & Fleeson, 2006). This uplifting effect should mitigate the negative effects of SA for extraverts but strengthen them for introverts as they do not experience these benefits from SA. Furthermore, the positive customer feedback should strengthen this relationship for extraverts even more (Côté, 2005) as they react more strongly to positive events than introverts. Consistent with the trait-congruency argument, SA should be less negative for extraverts because they are not as strongly affected by the physiological arousal prompted by response-focused ER (Demaree et al., 2004; Richards & Gross, 2006). There is evidence that introverts may respond less favorably to such arousal (Geen, 1984). | Judge et al. (2009) |
|  | The SA – emotional exhaustion relationship is partly mediated by state anxiety. | When employees react to ER by experiencing anxiety, it will draw upon his or her emotional resources (Baumeister et al., 1998). As the employee continues to experience anxiety over a given day, thereby continuing to draw upon his or her emotional resources, those resources become depleted, eventually resulting in a state of emotional exhaustion (Boyd et al., 2009). | Wagner et al. (2014) |
|  | The SA – emotional exhaustion relationship is partly mediated by NA (state). | State affect contributes to daily variations in job satisfaction and other indicators of employee well-being beyond the effects of trait affect (Ilies et al., 2007; Judge et al., 2006). Therefore, it might also influence emotional exhaustion. Furthermore, SA was found to influence NA (Richards & Gross, 2006; Robinson & Demaree, 2007) and a meta-analysis (Thoresen et al., 2003) found that only NA is a predictor of emotional exhaustion. | Judge et al. (2009) |
| Fatigue | SA (state) is negatively associated with fatigue (state). | ER, like any other effortful regulation, leads directly to the exhaustion of limited resources which are needed to continue behavior regulation and that fatigue is one indication of these resources diminishing(Baumeister et al., 1998; Muraven et al., 1998). SA leads to fatigue as a direct result of engaging in effortful self-control (Grandey, 2003; Muraven et al., 1998). | Beal et al. (2013) |
|  | The SA – fatigue relationship is partly mediated by strain (state). | Like the depletion that occurs through self-regulation, stressors and strains also are well-known contributors to fatigue (Lazarus & Folkman, 1984; Maslach et al., 2001). In addition to the direct effect of SA on fatigue, it also seems likely that the demands of SA can lead to the experience of strain, resulting in an indirect route from SA to fatigue through strain. | Beal et al. (2013) |
| Fatigue | The SA – fatigue relationship is moderated by affect spin (trait), with higher affect spin strengthening the relationship. | Ram and Gerstorf (2009) suggested individuals high in affect spin appear to react more strongly to events and conditions of their environment and are more reactive to affectively charged situations at work in comparison to those who are low in affect spin. SA at work represents a rather common affectively charged situation, and strain is one likely reaction to such situations. Consequently, as individuals high in affect spin have increased reactivity to affective events, they might therefore experience increased levels of strain reactions upon initiating this particularly taxing regulation strategy, finally resulting in the experience fatigue. | Beal et al. (2013) |
| Felt Authenticity | SA (state) negatively relates to felt authenticity (state).  DA (state) positively relates to felt authenticity (state). | From the discordance– congruence perspective of emotional labor (Mesmer-Magnus et al., 2012), the degree to which emotional displays are authentic is said to be the key difference between surface and deep acting. | Huppertz et al. (2020) |
| Insomnia | SA (state) is positively associated with insomnia (state). | Research indicates that stress and anxiety share part of the blame for sleep problems as they involve physiological arousal and sympathetic nervous system activation, which oppose the physiological processes involved in falling asleep (LeBlanc et al., 2009; Vahtera et al., 2007). As SA is thought to influence both anxiety and strain (Grandey, 2003; Lazarus & Folkman, 1984), it will probably result in insomnia. | Wagner et al. (2014) |
|  | The SA – insomnia relationship is partly mediated by state anxiety (state). | The higher levels of sympathetic activation (e.g., increased heart rate, constriction of blood vessels) relating to anxiety, tension and hyperarousal (Watson, 2000) might lead to difficulties sleeping as it interferes with calming down and relaxing (JrLeDuc et al., 2000; LeBlanc et al., 2009; Vahtera et al., 2007). | Wagner et al. (2014) |
| Job satisfaction | DA (state) is positively associated with job satisfaction (state). | DA might serve as a buffer against feeling fake and thus, the immediate effect could be a sense that one has provided service that is both authentic and consistent with the organization’s expectations (Hochschild, 1983). Furthermore, (Brotheridge & Grandey, 2002) found a positive relationship between DA and personal accomplishment, which might in turn positively influence job satisfaction. | Huang et al. (2015, pp. author-year ) and Judge et al. (2009) |
|  | The DA – job satisfaction relationship is moderated by extraversion (trait), with the relationship being stronger for extraverts than introverts. | Individuals experience more positive subjective outcomes when they behave in a manner consistent with their trait (Little, 2000; Moskowitz & Coté, 1995). Bono and Vey (2007) argued that behaving in the positive manner required by most customer service jobs should be uplifting for extraverts, who tend toward positive emotion (Lucas & Fujita, 2000; McNiel & Fleeson, 2006). As extraverts react more strongly to these positive effects, the relationship should be greater than for introverts. The positive customer feedback should strengthen this relationship for extraverts even more (Côté, 2005). Additionally, following self-verification theory (Swann & Read, 1981), extraverts may get feedback that confirms their views of themselves as outgoing and friendly, resulting in a more pleasant experience in comparison to disconfirmation of their self-perceptions. | Judge et al. (2009) |
| Job satisfaction | The DA – job satisfaction relationship is partly mediated by PA (state). | The experience of authentic positive emotions and the feelings of accomplishment stemming from DA are likely to lead employees to react more positively to their jobs (Zapf et al., 1999). The resulting PA might in turn influence job satisfaction (Thoresen et al., 2003). | Judge et al. (2009) |
|  | The DA – job satisfaction relationship is moderated by felt challenge (state), with higher felt challenge strengthening the relationship. | When perceiving their ongoing interactions as challenges, employees draw on resources to steer their emotional experiences toward the positive and away from the negative, while feeling a sense of accomplishment in doing so (Brotheridge & Grandey, 2002). When feeling challenged, agents are more likely to see personal resources at their disposal as exceeding situational demands (Blascovich & Tomaka, 1996), resulting in some kind of emotional reserve in the form of motivational resources. | Huang et al. (2015) |
|  | The conditional DA – felt challenge effect on job satisfaction is mediated by emotional exhaustion (state). | The availability of personal resources associated with reduced emotional exhaustion can lead employees to feel more positive toward their job (Cropanzano et al., 2003; Lee & Ashforth, 1990), perceiving the job as more rewarding and satisfying. Meanwhile, emotional exhaustion leads to unpleasant affect. Thus, emotionally exhausted employees may find it difficult to appreciate the positive aspects of their jobs and may try to distance themselves from their work, which is likely seen as the cause of exhaustion. | Huang et al. (2015) |
|  | SA (state) is negatively associated with job satisfaction (state). | SA negatively affects job satisfaction as it is at such moments when conflicts between one’s own needs and preferences and the job’s demands are most salient and job dissatisfaction highest (Grandey, 2000; Hochschild, 1983). | Judge et al. (2009) and Bono et al. (2007) |
|  | The SA – job satisfaction relationship is partly mediated by NA (state). | State affect contributes to daily variations in job satisfaction and other indicators of employee well-being beyond the effects of trait affect (Ilies et al., 2007; Niklas & Dormann, 2005). A meta-analysis by Thoresen et al. (2003) found that job satisfaction is influenced by PA and NA. | Judge et al. (2009) |
|  | The SA – job satisfaction relationship is moderated by extraversion (trait), with the relationship being stronger for introverts. | Individuals experience more positive subjective outcomes when they behave in a manner consistent with their trait (Little, 2000; Moskowitz & Coté, 1995). Bono and Vey (2007) argued that behaving in the positive manner required by most customer service jobs should be uplifting for extraverts, who tend toward positive emotion (Fleeson et al., 2002; Lucas & Fujita, 2000; McNiel & Fleeson, 2006). This uplifting effect should weaken the negative effects of SA for extraverts. Furthermore, the positive customer feedback should counteract the negative relationship between SA and job satisfaction for extraverts (Côté, 2005) and as extraverts react more strongly to these positive effects, the influence should be even greater. | Judge et al. (2009) |
| Job satisfaction | The SA – job satisfaction relationship is moderated by the supervisors’ transformational leadership behaviors (trait), with less transformational leadership strengthening the relationship. | Bono and Judge (2003) demonstrated that managers’ transformational leadership behaviors can influence employees’ identification with their work. They also found that transformational leadership was a positive predictor of the extent to which employees felt that their work activities were self-congruent and consistent with their own interests and values. In line with self-determination theory (Ryan & Deci, 2000) and the goal self-concordance model (Sheldon & Elliot, 1999) there are links between authentic self-expression and individual well-being. Accordingly, Ashforth and Humphrey (1993) suggested that employees who identify with their work are more likely to feel authentic even when conforming to role expectations as e.g. ER demands. | Bono et al. (2007) |
| Mental health | SA (state) is negatively associated with employees’ mental health (contentment, resilience, and peace of mind; state). | According to the Occupational Stress Indicator (Lu et al., 2003), intense stress will have a negative influence on an individual’s mental and physical health (Cooper et al., 2001). Furthermore, SA often leads to serious psychological problems, such as emotional disorders, emotional exhaustion, depersonalization, and low job satisfaction (Chi & Liang, 2013). | Xiao et al. (2019) |
|  | The SA – mental health relationship is moderated by negative group emotional contagion (trait), with more negative group emotional contagion strengthening the relationship. | The social information processing theory proposes that employees are influenced by the emotions and attitudes of others in an organizational context (Chiu et al., 2016). Because negative group emotional contagion will lead to team members feeling unhelpful, frustrated and depressed, it is more likely that they will suppress their real emotions (Roberts et al., 2015). | Xiao et al. (2019) |
|  | The SA – mental health relationship is moderated by positive group emotional contagion (trait), with more positive group emotional contagion weakening the relationship. | The job demands–resources model suggests that external resources (e.g., supervisor support) may trigger a motivational process and enhance job engagement, job-related learning, and organizational commitment (Demerouti et al., 2001; Taris & Feij, 2004). If employees are supported by colleagues or by organizational support systems, such as mentoring or career advice, this may reduce the negative impact of SA (Taris & Feij, 2004). | Xiao et al. (2019) |
| Mood (NA/PA) | DA (state) is negatively associated with NA (state). | DA may change employees’ perceptions of their (negative) emotions (Grandey, 2000; Gross, 1998). With DA, individuals may deploy their attention elsewhere by conjuring thoughts to elicit desired affective states, or they may change their cognitive perspective by reappraising their situation, resulting in emotions that are commensurate with display rules (Grandey, 2000; Hochschild, 1983). Therefore, DA should decrease NA, which is generally rejected by customer service display rules. | Scott and Barnes (2011) |
|  | The DA – NA relationship is moderated by gender (trait), with the relationship being stronger for females than males. | EL relationships may vary by gender, in that women may be better at managing emotions, but they would be engaging in more suppression of true feelings (Grandey, 2000). Thus, women should experience more NA when engaging in DA. | Scott and Barnes (2011) |
| Mood (NA/PA) | DA (state) is positively associated to PA (state). | With DA, individuals try to align their emotions to the integrative display rules, i.e. the preference of positive over negative emotions (Grandey, 2000; Gross, 1998; Hochschild, 1983). Some of the relating strategies as e.g. distraction seeking, reappraisal of the situation, and evocation of memories can be effective in inducing positive moods (Joormann et al., 2007). | (Judge et al., 2009) and Scott and Barnes (2011) |
|  | The DA – PA relationship is moderated by extraversion (trait), with the relationship being stronger for extraverts than introverts. | Individuals experience more positive subjective outcomes when they behave in a manner consistent with their trait (Little, 2000; Moskowitz & Coté, 1995). Bono and Vey (2007) argued that behaving in the positive manner required by most customer service jobs should be uplifting for extraverts, who tend toward positive emotion (Fleeson et al., 2002; Lucas & Fujita, 2000; McNiel & Fleeson, 2006). This uplifting effect should strengthen the positive effects of DA for extraverts. Additionally, the positive customer feedback should amplify this negative relationship for extraverts even more (Côté, 2005). Furthermore, following self-verification theory (Swann & Read, 1981), extraverts may get feedback that confirms their views of themselves as outgoing and friendly, resulting in the unpleasant feeling that accompanies self-verification. | Judge et al. (2009) |
|  | The DA – PA relationship is moderated by gender (trait), with the relationship being stronger for females than males. | EL relationships may vary by gender, in that women may be better at managing emotions, but they would be engaging in more suppression of true feelings (Grandey, 2000). Likewise, Brody and Hall (2008) found that women are both expected to and do show greater emotional intensity and emotional expressiveness than men, and such differences hold for both positive and negative emotions, so the positive relationship between DA and PA should be stronger for females than males. | Scott and Barnes (2011) |
|  | SA (state) is positively associated with NA (state). | SA does nothing to change underlying negative emotions (Grandey, 2000; Gross, 1998; Hochschild, 1983), so it is likely that the negative emotions are preserved. As a matter of fact, those affective states may actually worsen as a result of this EL strategy. Research has shown that attempting to suppress negative emotions may have the ironic effect of causing individuals to think about the eliciting situation even more, which ultimately intensifies those negative feelings (Wegner, 1994). Furthermore, the suppression, exaggeration and faking of emotions was found to increase physiological arousal (Richards & Gross, 2006; Robinson & Demaree, 2007), which is interpreted negatively in the absence of other information (Schachter & Singer, 1962). | Scott and Barnes (2011) and Judge et al. (2009) |
| Mood (NA/PA) | The SA – NA relationship is moderated by extraversion (trait), with the relationship being stronger for introverts than extraverts. | Individuals experience more positive subjective outcomes when they behave in a manner consistent with their trait (Little, 2000; Moskowitz & Coté, 1995). Bono and Vey (2007) argued that behaving in the positive manner required by most customer service jobs should be uplifting for extraverts, who tend toward positive emotion (Fleeson et al., 2002; Lucas & Fujita, 2000; McNiel & Fleeson, 2006). This uplifting effect should weaken the negative effects of SA for extraverts. The positive customer feedback should also counteract this negative relationship for extraverts (Côté, 2005). Furthermore, following self-verification theory (Swann & Read, 1981), extraverts may get feedback that confirms their views of themselves as outgoing and friendly, somewhat offsetting the dissonance created by behavior that is at variance with the underlying feelings. This feedback is contrary to introverts’ personality and such they might react more negatively to it (Swann, 2005). | Judge et al. (2009) |
|  | The SA – NA relationship is moderated by gender (trait), with the relationship being stronger for females than males. | EL relationships may vary by gender, in that women may be better at managing emotions, but they would be engaging in more suppression of true feelings (Grandey, 2000). Likewise, Brody and Hall (2008) found that women are both expected to and do show greater emotional intensity and emotional expressiveness than men, and such differences hold for both positive and negative emotions, so the positive relationship between SA and NA should be stronger for females than males. | Scott and Barnes (2011) |
|  | SA (state) is negatively associated with PA (state). | SA does nothing to change underlying emotions (Grandey, 2000; Gross, 1998; Hochschild, 1983) and thus does not enhance positive emotions. As a matter of fact, those affective states may actually worsen as a result of this EL strategy (Wegner, 1994). Furthermore, the suppression, exaggeration and faking of emotions was found to increase physiological arousal (Richards & Gross, 2006; Robinson & Demaree, 2007),, which is interpreted negatively in the absence of other information (Schachter & Singer, 1962). | Scott and Barnes (2011) |
|  | The SA – PA relationship is moderated by gender (trait), with the relationship being stronger for females than males. | EL relationships may vary by gender, in that women may be better at managing emotions, but they would be engaging in more suppression of true feelings (Grandey, 2000). Likewise, Brody and Hall (2008) found that women are both expected to and do show greater emotional intensity and emotional expressiveness than men, and such differences hold for both positive and negative emotions, so the positive relationship between SA and PA should be stronger for females than males. | Scott and Barnes (2011) |
| Psychological Effort | SA and DA (state) positively relate to psychological effort (state). | Psychological effort is an important mechanism proposed in the emotional labor literature to explain relationships of emotional labor strategies with well-being and strain. A central tenet of the emotional labor literature is that surface acting consumes more resources and is therefore more effortful than deep acting (Goldberg & Grandey, 2007; Totterdell & Holman, 2003). | Huppertz et al. (2020) |
|  | The positive relationship between SA and psychological effort is stronger than the one between DA and psychological effort. |  |  |
| Rewarding Interactions | SA (state) negatively relates to rewarding interactions (state).  DA (state) positively relates to rewarding interactions (state). | From the perspective of the social interaction model of emotional labor (Côté, 2005), the quality of social interactions is key to understanding the effects of surface and deep acting on employee strain. | Huppertz et al. (2020) |
| Service performance | DA (state) will be more positively associated with service performance (state) than SA (state). | ER leads to the expression of affective states that conform to display rules, and the way in which employees manage their feelings and expressions can influence the effectiveness of their interactions with customers and thus play an important role in influencing customers to purchase a product, to remain loyal to the organization, or to tell others about the service given (Hochschild, 1983). Generally, employees amplify positive emotions during customer service to facilitate service interactions and foster positive social responses from customers (Côté & Morgan, 2002; Darley & Fazio, 1980). Additionally, (Grandey, 2003) found SA being negatively associated, but DA being positively associated with effective emotional displays during customer interactions. | Totterdell and Holman (2003) |
| Strain-based work-to-family conflict | SA (state) is positively associated with strain-based work-to-family conflict (state). | Conflicts arising from workplace stressors can make it difficult for an employee to be fully engaged in home life and family roles(Carlson et al., 2000). Given the permeability of many employees’ boundaries between work and home domains (Ashforth et al., 2000), it is fairly possible that the strain at work generated from SA is likely to persist even if the employee comes home (Ilies et al., 2007). This spillover process is consistent with models of work–family conflict(Edwards & Rothbard, 2000; Yanchus et al., 2010) and with research showing that negative work emotions mediate the influence of work demands on home emotions and work-to-family conflict (Ilies et al., 2007). | Wagner et al. (2014) |
|  | The SA – strain-based work-to-family conflict relationship is partly mediated by state anxiety. | Anxiety could be such a discrete emotion through which the demands present in EL could influence work-to-family conflict. | Wagner et al. (2014) |
| Stress/strain | SA (state) is positively associated with experienced stress/strain (state). | Theoretical assumptions include that EL creates a sense of strain (Hochschild, 1983). An experimental examination also showed that individuals in an emotion suppression condition experienced higher levels of sympathetic activation, which is an indicator of stress (e.g., increased heart rate, constriction of blood vessels) than participants who were not instructed to suppress their emotions (Gross, 1998). Furthermore, SA should elicit more negative reactions from customers because it is inauthentic and this feedback leads to strain for the employee (Côté, 2005) | Bono et al. (2007) and Beal et al. (2013) |
| Stress/strain | The SA – stress relationship is moderated by supervisors’ transformational leadership behaviors (trait), with less transformational leadership behavior strengthening the relationship. | Bono and Judge (2003) demonstrated that managers’ transformational leadership behaviors can influence employees’ identification with their work. They also found that transformational leadership was a positive predictor of the extent to which employees felt that their work activities were self-congruent and consistent with their own interests and values. In line with Self-determination theory (Ryan & Deci, 2000) and the goal self-concordance model(Sheldon & Elliot, 1999) there are links between authentic self-expression and individual well-being. Accordingly, Ashforth and Humphrey (1993) suggested that employees who identify with their work are more likely to feel authentic even when conforming to role expectations as e.g. ER demands. This might weaken the negative effects of SA on stress. | Bono et al. (2007) |
|  | The SA – strain relationship is moderated by affect spin (trait), with higher affect spin strengthening the relationship. | Ram and Gerstorf (2009) suggested that individuals high in affect spin appear to react more strongly to events and conditions of their environment and are more reactive to affectively charged situations at work in comparison to those who are low in affect spin. SA at work represents a rather common affectively charged situation, and strain is one likely reaction to such situations. Consequently, as individuals high in affect spin have increased reactivity to affective events, they might experience increased levels of strain reactions upon initiating this particularly taxing regulation strategy. | Beal et al. (2013) |
| Work withdrawal | DA (state) is negatively associated with perceptions of work withdrawal (state). | Grandey (2000) specified links between EL and work withdrawal. Employees may utilize work withdrawal as a form of rest, allowing them to return to self-regulatory demands of EL with renewed strength (Muraven & Baumeister, 2000). But as DA is also associated with benefits for the employee as e.g. positive social feedback(Côté, 2005; Darley & Fazio, 1980), it might mitigate the feeling of needing rest. | Scott and Barnes (2011) |
|  | The DA – work withdrawal relationship is moderated by gender (trait), with the relationship being stronger for females than males. | EL relationships may vary by gender, in that women may be better at managing emotions, but they would be also engaging in more suppression of true feelings (Grandey, 2000). The generation of positive affective states via DA should not only be more role-appropriate for women than for men, but also, to the extent that women experience such states more intensely (Brody & Hall, 2008), DA should yield a greater gain for women than for men. In addition, research has shown that women are more adept than men at producing “authentic” smiles (Merten, 1997), implying that women are better at DA than men. | Scott and Barnes (2011) |
|  | The DA – work withdrawal relationship is partly mediated by NA (state). | To the extent that DA produces a change in affect for the better (i.e., decreased NA), it should be negatively associated with work withdrawal. | Scott and Barnes (2011) |
| Work withdrawal | The conditional DA – NA effect on work withdrawal is moderated by gender (trait), with the effect being stronger for females than for males. | EL relationships may vary by gender, in that women may be better at managing emotions, but they would be engaging in more suppression of true feelings (Grandey, 2000). As women show greater emotional intensity and emotional expressiveness than men, and such differences hold for both positive and negative emotions (Brody & Hall, 2008), women should experience higher NA than men and thus a stronger conditional DA – NA effect on work withdrawal. | Scott and Barnes (2011) |
|  | The DA – work withdrawal relationship is partly mediated by PA (state). | To the extent that DA produces a change in affect for the better (i.e., increased PA), DA should be negatively associated with work withdrawal.  Positive affective states prompt approach rather than avoidance behavior by stimulating creativity and search as well as by expanding individuals’ action repertoires (Cacioppo et al., 1999; Fredrickson, 2001). Given that individuals experiencing positive affective states engage their environments, it follows that, in work contexts, employees should report lower levels of work withdrawal on days when they experience PA. | Scott and Barnes (2011) |
|  | The conditional DA – PA effect on work withdrawal is moderated by gender (trait), with the effect being stronger for females than for males. | EL relationships may vary by gender, in that women may be better at managing emotions, but they would also be engaging in more suppression of true feelings (Grandey, 2000) | Scott and Barnes (2011) |
|  | SA (state) is positively associated with work withdrawal (state). | (Grandey, 2000) specified links between EL and work withdrawal. Employees may utilize work withdrawal as a form of rest, allowing them to return to self-regulatory demands of EL with renewed strength (Muraven & Baumeister, 2000). Willpower is fatigued after exertion, making one less able to perform self-control acts until strength is recovered (Baumeister et al., 2007). | Scott and Barnes (2011) |
|  | The SA – work withdrawal relationship is moderated by gender (trait), with the relationship being stronger for females than males. | EL relationships may vary by gender, in that women may be better at managing emotions, but they would be engaging in more suppression of true feelings (Grandey, 2000). As women show greater emotional intensity and emotional expressiveness than men, and such differences hold for both positive and negative emotions (Brody & Hall, 2008), women should experience higher PA than men and thus also a stronger conditional DA – PA effect on work withdrawal. | Scott and Barnes (2011) |
|  | The SA – work withdrawal relationship is partly mediated by NA (state). | To the extent that SA produces a change in affect for the worse (i.e., increased NA), SA should be positively associated with work withdrawal. As Elfenbein (2007) noted, the action tendencies of negative affective states address current problems to improve the situation, which is accomplished through avoidance (as opposed to approach) in either the short or long term (Cacioppo et al., 1999). Applied to the work context, the notion of action tendencies implies that employees should report higher levels of work withdrawal on days in which they experience NA. | Scott and Barnes (2011) |
| Work withdrawal | The conditional SA – NA effect on work withdrawal is moderated by gender (state), with the effect being stronger for females than for males. | EL relationships may vary by gender, in that women may be better at managing emotions, but they would be engaging in more suppression of true feelings (Grandey, 2000). As women show greater emotional intensity and emotional expressiveness than men, and such differences hold for both positive and negative emotions (Brody & Hall, 2008), women should experience higher NA than men and thus also a stronger conditional SA – NA effect on work withdrawal. | Scott and Barnes (2011) |
|  | The SA – work withdrawal relationship is partly mediated by PA (state). | To the extent that SA produces a change in affect for the worse (i.e., decreased PA), SA should be positively associated with work withdrawal. Positive affective states prompt approach rather than avoidance behavior by stimulating creativity and search as well as by expanding individuals’ action repertoires (Cacioppo et al., 1999; Fredrickson, 2001). Given that individuals experiencing positive affective states engage their environments, it follows that, in work contexts, employees should report lower levels of work withdrawal on days when they experience PA. | Scott and Barnes (2011) |
|  | The conditional SA – PA effect on work withdrawal is moderated by gender (trait), with the effect being stronger for females than for males. | EL relationships may vary by gender, in that women may be better at managing emotions, but they would be engaging in more suppression of true feelings (Grandey, 2000). As women show greater emotional intensity and emotional expressiveness than men, and such differences hold for both positive and negative emotions (Brody & Hall, 2008), women should experience higher PA than men and thus also a stronger conditional SA – PA effect on work withdrawal. | Scott and Barnes (2011) |
| *Note*. EL = Emotional Labor. ER = Emotion Regulation. SA = SA. DA = DA. NA = Negative Affect. PA = Positive Affect. | | | |

**References**

Ashforth, B. E., & Humphrey, R. H. (1993). Emotional Labor in Service Roles: The Influence of Identity. *The Academy of Management Review*, *18*(1), 88. https://doi.org/10.2307/258824

Ashforth, B. E., Kreiner, G. E., & Fugate, M. (2000). All in a day's work: Boundaries and micro role transitions. *Academy of Management Review*, *25*(3), 472-491. https://doi.org/Doi 10.2307/259305

Baumeister, R. F., Bratslavsky, E., Muraven, M., & Tice, D. M. (1998). Ego Depletion: Is the Active Self a Limited Resource? *Journal of Personality and Social Psychology*, *74*(5), 1252–1265.

Baumeister, R. F., Tice, D. M., & Vohs, K. D. (2018). The Strength Model of Self-Regulation: Conclusions From the Second Decade of Willpower Research. *Perspect Psychol Sci*, *13*(2), 141-145. https://doi.org/10.1177/1745691617716946

Baumeister, R. F., & Vohs, K. D. (2016). Strength Model of Self-Regulation as Limited Resource. In M. P. Zanna & J. M. Olson (Eds.), *Advances in Experimental Social Psychology* (Vol. 54, pp. 67–127). Elsevier Science. https://doi.org/10.1016/bs.aesp.2016.04.001

Baumeister, R. F., Vohs, K. D., & Tice, D. M. (2007). The strength model of self-control. *CURRENT DIRECTIONS IN PSYCHOLOGICAL SCIENCE*, *16*(6), 351-355. https://doi.org/DOI 10.1111/j.1467-8721.2007.00534.x

Beal, D. J., Trougakos, J. P., Weiss, H. M., & Dalal, R. S. (2013). Affect spin and the emotion regulation process at work. *J Appl Psychol*, *98*(4), 593-605. https://doi.org/10.1037/a0032559

Blascovich, J., & Tomaka, J. (1996). The Biopsychosocial Model of Arousal Regulation. In M. P. Zanna (Ed.), *Advances in Experimental Social Psychology Volume 28* (Vol. 28, pp. 1-51). Academic Press. https://doi.org/10.1016/s0065-2601(08)60235-x

Bono, J. E., Foldes, H. J., Vinson, G., & Muros, J. P. (2007). Workplace emotions: the role of supervision and leadership. *J Appl Psychol*, *92*(5), 1357-1367. https://doi.org/10.1037/0021-9010.92.5.1357

Bono, J. E., & Judge, T. A. (2003). Self-concordance at work: Toward understanding the motivational effects of transformational leaders. *ACADEMY OF MANAGEMENT JOURNAL*, *46*(5), 554-571. https://doi.org/Doi 10.2307/30040649

Bono, J. E., & Vey, M. A. (2007). Personality and emotional performance: extraversion, neuroticism, and self-monitoring. *J Occup Health Psychol*, *12*(2), 177-192. https://doi.org/10.1037/1076-8998.12.2.177

Boyd, N. G., Lewin, J. E., & Sager, J. K. (2009). A model of stress and coping and their influence on individual and organizational outcomes. *JOURNAL OF VOCATIONAL BEHAVIOR*, *75*(2), 197-211. https://doi.org/https://doi.org/10.1016/j.jvb.2009.03.010

Brody, L. R., & Hall, J. A. (2008). Gender and emotion in context. In M. Lewis, J. M. Haviland-Jones, & L. F. Barrett (Eds.), *Handbook of emotions, 3rd ed* (pp. 395–408). The Guilford Press.

Brotheridge, C. M., & Grandey, A. A. (2002). Emotional labor and burnout: Comparing two perspectives of "people work". *JOURNAL OF VOCATIONAL BEHAVIOR*, *60*(1), 17-39. https://doi.org/10.1006/jvbe.2001.1815

Brotheridge, C. M., & Lee, R. T. (2002). Testing a conservation of resources model of the dynamics of emotional labor. *Journal of occupational health psychology*, *7*(1), 57-67. https://doi.org/10.1037/1076-8998.7.1.57

Cacioppo, J. T., Gardner, W. L., & Berntson, G. G. (1999). The affect system has parallel and integrative processing components: Form follows function. *Journal of Personality and Social Psychology*, *76*(5), 839-855. https://doi.org/Doi 10.1037/0022-3514.76.5.839

Callaghan, G., & Thompson, P. (2002). 'We recruit attitude': The selection and shaping of routine call centre labour. *Journal of Management Studies*, *39*(2), 233-254. https://doi.org/Doi 10.1111/1467-6486.00290

Carlson, D. S., Kacmar, K. M., & Williams, L. J. (2000). Construction and initial validation of a multidimensional measure of work-family conflict. *JOURNAL OF VOCATIONAL BEHAVIOR*, *56*(2), 249-276. https://doi.org/DOI 10.1006/jvbe.1999.1713

Chang, M. L. (2013). Toward a theoretical model to understand teacher emotions and teacher burnout in the context of student misbehavior: Appraisal, regulation and coping. *Motivation and Emotion*, *37*(4), 799-817. https://doi.org/10.1007/s11031-012-9335-0

Chang, M. L., & Taxer, J. (2021). Teacher emotion regulation strategies in response to classroom misbehavior [Article]. *Teachers and Teaching*, *27*(5), 353-369. https://doi.org/10.1080/13540602.2020.1740198

Chi, S.-C. S., & Liang, S.-G. (2013). When do subordinates' emotion-regulation strategies matter? Abusive supervision, subordinates' emotional exhaustion, and work withdrawal. *The Leadership Quarterly*, *24*(1), 125-137. https://doi.org/10.1016/j.leaqua.2012.08.006

Chiu, C. C., Owens, B. P., & Tesluk, P. E. (2016). Initiating and utilizing shared leadership in teams: The role of leader humility, team proactive personality, and team performance capability. *J Appl Psychol*, *101*(12), 1705-1720. https://doi.org/10.1037/apl0000159

Cooper, C. L., Dewe, P., & O'Driscoll, M. (2001). *Organizational Stress: A Review and Critique of Theory, Research, and Applications*. Sage. https://doi.org/10.4135/9781452231235

Côté, S. (2005). A Social Interaction Model Of The Effects Of Emotion Regulation On Work Strain. *Academy of Management Review*, *30*(3), 509-530. https://doi.org/10.5465/amr.2005.17293692

Côté, S., & Morgan, L. M. (2002). A longitudinal analysis of the association between emotion regulation, job satisfaction, and intentions to quit. *Journal of Organizational Behavior*, *23*(8), 947-962. https://doi.org/10.1002/job.174

Cropanzano, R., Rupp, D. E., & Byrne, Z. S. (2003). The relationship of emotional exhaustion to work attitudes, job performance, and organizational citizenship behaviors. *J Appl Psychol*, *88*(1), 160-169. https://doi.org/10.1037/0021-9010.88.1.160

Darley, J. M., & Fazio, R. H. (1980). Expectancy confirmation processes arising in the social interaction sequence. *American Psychologist*, *35*(10), 867-881. https://doi.org/10.1037/0003-066x.35.10.867

Demaree, H. A., Schmeichel, B. J., Robinson, J. L., & Everhart, D. E. (2004). Behavioural, affective, and physiological effects of negative and positive emotional exaggeration. *Cognition & Emotion*, *18*(8), 1079-1097. https://doi.org/10.1080/02699930441000085

Demerouti, E., Bakker, A. B., Nachreiner, F., & Schaufeli, W. B. (2001). The job demands-resources model of burnout. *J Appl Psychol*, *86*(3), 499-512. https://www.ncbi.nlm.nih.gov/pubmed/11419809

Edwards, J. R., & Rothbard, N. P. (2000). Mechanisms linking work and family: Clarifying the relationship between work and family constructs. *Academy of Management Review*, *25*(1), 178-199. https://doi.org/Doi 10.2307/259269

Elfenbein, H. A. (2007). 7 Emotion in Organizations. *Academy of Management Annals*, *1*(1), 315-386. https://doi.org/10.5465/078559812

Fleeson, W., Malanos, A. B., & Achille, N. M. (2002). An intraindividual process approach to the relationship between extraversion and positive affect: Is acting extraverted as "good" as being extraverted? *Journal of Personality and Social Psychology*, *83*(6), 1409-1422. https://doi.org/10.1037/0022-3514.83.6.1409

Fredrickson, B. L. (2001). The role of positive emotions in positive psychology. The broaden-and-build theory of positive emotions. *Am Psychol*, *56*(3), 218-226. https://doi.org/10.1037//0003-066x.56.3.218

Geen, R. G. (1984). Preferred stimulation levels in introverts and extroverts: Effects on arousal and performance. *Journal of Personality and Social Psychology*, *46*(6), 1303-1312. https://doi.org/10.1037/0022-3514.46.6.1303

Goldberg, L. S., & Grandey, A. A. (2007). Display rules versus display autonomy: emotion regulation, emotional exhaustion, and task performance in a call center simulation. *J Occup Health Psychol*, *12*(3), 301-318. https://doi.org/10.1037/1076-8998.12.3.301

Grandey, A. A. (2000). Emotion regulation in the workplace: a new way to conceptualize emotional labor. *J Occup Health Psychol*, *5*(1), 95-110. https://doi.org/10.1037//1076-8998.5.1.95

Grandey, A. A. (2003). When "the show must go on": Surface acting and deep acting as determinants of emotional exhaustion and peer-rated service delivery. *ACADEMY OF MANAGEMENT JOURNAL*, *46*(1), 86-96. https://doi.org/10.2307/30040678

Grandey, A. A., Frone, M. R., Melloy, R. C., & Sayre, G. M. (2019). When are fakers also drinkers? A self-control view of emotional labor and alcohol consumption among U.S. service workers. *J Occup Health Psychol*, *24*(4), 482-497. https://doi.org/10.1037/ocp0000147

Grandey, A. A., & Gabriel, A. S. (2015). Emotional Labor at a Crossroads: Where Do We Go from Here? *Annual Review of Organizational Psychology and Organizational Behavior, Vol 2*, *2*, 323-349. https://doi.org/10.1146/annurev-orgpsych-032414-111400

Gross, J. J. (1998). The Emerging Field of Emotion Regulation: An Integrative Review. *Review of General Psychology*, *2*(3), 271–299.

Gross, J. J. (2015). Emotion Regulation: Current Status and Future Prospects. *Psychological Inquiry*, *26*(1), 1-26. https://doi.org/10.1080/1047840x.2014.940781

Gross, J. J., & Thompson, R. A. (2007). Emotion Regulation: Conceptual Foundations. In J. J. Gross (Ed.), *Handbook of emotion regulation.* (pp. 3-24). Guilford Press.

Hochschild, A. R. (1983). *The Managed Heart: Commercialization of Human Feeling*. University of California Press.

Huang, J. L., Chiaburu, D. S., Zhang, X. A., Li, N., & Grandey, A. A. (2015). Rising to the challenge: Deep acting is more beneficial when tasks are appraised as challenging. *J Appl Psychol*, *100*(5), 1398-1408. https://doi.org/10.1037/a0038976

Hulsheger, U. R., & Schewe, A. F. (2011). On the Costs and Benefits of Emotional Labor: A Meta-Analysis of Three Decades of Research. *Journal of occupational health psychology*, *16*(3), 361-389. https://doi.org/10.1037/a0022876

Huppertz, A. V., Hulsheger, U. R., Velozo, J. D., & Schreurs, B. H. (2020). Why Do Emotional Labor Strategies Differentially Predict Exhaustion? Comparing Psychological Effort, Authenticity, and Relational Mechanisms [Article]. *Journal of occupational health psychology*, *25*(3), 214-226. https://doi.org/10.1037/ocp0000179

Ilies, R., Schwind, K. M., & Heller, D. (2007). Employee well-being: A multilevel model linking work and nonwork domains. *European Journal of Work and Organizational Psychology*, *16*(3), 326-341. https://doi.org/10.1080/13594320701363712

Inzlicht, M., & Schmeichel, B. J. (2012). What Is Ego Depletion? Toward a Mechanistic Revision of the Resource Model of Self-Control. *Perspect Psychol Sci*, *7*(5), 450-463. https://doi.org/10.1177/1745691612454134

Inzlicht, M., Schmeichel, B. J., & Macrae, C. N. (2014). Why self-control seems (but may not be) limited. *Trends Cogn Sci*, *18*(3), 127-133. https://doi.org/10.1016/j.tics.2013.12.009

Joormann, J., Siemer, M., & Gotlib, I. H. (2007). Mood regulation in depression: Differential effects of distraction and recall of happy memories on sad mood. *J Abnorm Psychol*, *116*(3), 484-490. https://doi.org/10.1037/0021-843X.116.3.484

JrLeDuc, P. A., Caldwell, J. A., Jr., & Ruyak, P. S. (2000). The effects of exercise as a countermeasure for fatigue in sleep-deprived aviators. *Mil Psychol*, *12*(4), 249-266. https://doi.org/10.1207/S15327876MP1204_02

Judge, T. A., Scott, B. A., & Ilies, R. (2006). Hostility, job attitudes, and workplace deviance: test of a multilevel model. *J Appl Psychol*, *91*(1), 126-138. https://doi.org/10.1037/0021-9010.91.1.126

Judge, T. A., Woolf, E. F., & Hurst, C. (2009). Is Emotional Labor More Difficult for Some Than for Others? A Multilevel, Experience-Sampling Study. *PERSONNEL PSYCHOLOGY*, *62*(1), 57-88. https://doi.org/10.1111/j.1744-6570.2008.01129.x

Keller, M. M., Chang, M. L., Becker, E. S., Goetz, T., & Frenzel, A. C. (2014). Teachers' emotional experiences and exhaustion as predictors of emotional labor in the classroom: an experience sampling study. *Frontiers in psychology*, *5*. https://doi.org/10.3389/fpsyg.2014.01442

Lazarus, R. S., & Folkman, S. (1984). *Stress, Appraisal, and Coping*. Springer Publishing Company, Inc.

LeBlanc, M., Merette, C., Savard, J., Ivers, H., Baillargeon, L., & Morin, C. M. (2009). Incidence and risk factors of insomnia in a population-based sample. *Sleep*, *32*(8), 1027-1037. https://doi.org/10.1093/sleep/32.8.1027

Lee, M., Pekrun, R., Taxer, J. L., Schutz, P. A., Vogl, E., & Xie, X. (2016). Teachers’ emotions and emotion management: integrating emotion regulation theory with emotional labor research. *Social Psychology of Education*, *19*(4), 843-863. https://doi.org/10.1007/s11218-016-9359-5

Lee, R. T., & Ashforth, B. E. (1990). On the meaning of Maslach's three dimensions of burnout. *J Appl Psychol*, *75*(6), 743-747. https://doi.org/10.1037/0021-9010.75.6.743

Lee, R. T., & Ashforth, B. E. (1996). A meta-analytic examination of the correlates of the three dimensions of job burnout. *J Appl Psychol*, *81*(2), 123-133. https://doi.org/10.1037/0021-9010.81.2.123

Little, B. R. (2000). Free traits and personal contexts: Expanding a social ecological model of well-being. In W. B. Walsh, K. H. Craik, & R. H. Price (Eds.), *Person–environment psychology: New directions and perspectives* (2 ed., pp. 87–116). Lawrence Erlbaum Associates Publishers.

Lu, L., Cooper, C. L., Kao, S. F., & Zhou, Y. (2003). Work stress, control beliefs and well‐being in Greater China. *Journal of Managerial Psychology*, *18*(6), 479-510. https://doi.org/10.1108/02683940310494359

Lucas, R. E., & Fujita, F. (2000). Factors influencing the relation between extraversion and pleasant affect. *Journal of Personality and Social Psychology*, *79*(6), 1039-1056. https://doi.org/10.1037//0022-3514.79.6.1039

Maslach, C., Schaufeli, W. B., & Leiter, M. P. (2001). Job burnout. *Annual review of psychology*, *52*, 397-422. https://doi.org/DOI 10.1146/annurev.psych.52.1.397

McNiel, J. M., & Fleeson, W. (2006). The causal effects of extraversion on positive affect and neuroticism on negative affect: Manipulating state extraversion and state neuroticism in an experimental approach. *Journal of Research in Personality*, *40*(5), 529-550. https://doi.org/10.1016/j.jrp.2005.05.003

Merten, J. (1997). Facial-affective behavior, mutual gaze, and emotional experience in dyadic interactions. *Journal of Nonverbal Behavior*, *21*(3), 179-201. https://doi.org/Doi 10.1023/A:1024969514170

Mesmer-Magnus, J. R., DeChurch, L. A., & Wax, A. (2012). Moving emotional labor beyond surface and deep acting: A discordance-congruence perspective. *Organizational Psychology Review*, *2*(1), 6-53. https://doi.org/10.1177/2041386611417746

Morris, J. A., & Feldman, D. C. (1996). The dimensions, antecedents, and consequences of emotional labor. *Academy of Management Review*, *21*(4), 986-1010. https://doi.org/Doi 10.2307/259161

Moskowitz, D. S., & Coté, S. (1995). Do interpersonal traits predict affect? A comparison of three models. *Journal of Personality and Social Psychology*, *69*(5), 915-924. https://doi.org/10.1037/0022-3514.69.5.915

Muraven, M., & Baumeister, R. F. (2000). Self-regulation and depletion of limited resources: Does self-control resemble a muscle? *Psychological Bulletin*, *126*(2), 247-259. https://doi.org/10.1037//0033-2909.126.2.247

Muraven, M., & Shmueli, D. (2006). The self-control costs of fighting the temptation to drink. *Psychol Addict Behav*, *20*(2), 154-160. https://doi.org/10.1037/0893-164X.20.2.154

Muraven, M., Tice, D. M., & Baumeister, R. F. (1998). Self-control as limited resource: Regulatory depletion patterns. *Journal of Personality and Social Psychology*, *74*(3), 774-789. https://doi.org/Doi 10.1037/0022-3514.74.3.774

Näring, G., Briët, M., & Brouwers, A. (2006). Beyond demand–control: Emotional labour and symptoms of burnout in teachers. *Work & Stress*, *20*(4), 303-315. https://doi.org/10.1080/02678370601065182

Niklas, C. D., & Dormann, C. (2005). The impact of state affect on job satisfaction. *European Journal of Work and Organizational Psychology*, *14*(4), 367-388. https://doi.org/10.1080/13594320500348880

Palmatier, R. W., Dant, R. R., Grewal, D., & Evans, K. R. (2006). Factors influencing the effectiveness of relationship marketing: A meta-analysis. *Journal of Marketing*, *70*(4), 136-153. https://doi.org/DOI 10.1509/jmkg.70.4.136

Ram, N., & Gerstorf, D. (2009). Time-structured and net intraindividual variability: tools for examining the development of dynamic characteristics and processes. *Psychol Aging*, *24*(4), 778-791. https://doi.org/10.1037/a0017915

Richards, J. M., & Gross, J. J. (2006). Personality and emotional memory: How regulating emotion impairs memory for emotional events. *Journal of Research in Personality*, *40*(5), 631-651. https://doi.org/10.1016/j.jrp.2005.07.002

Roberts, S., O'Connor, K., Aardema, F., & Belanger, C. (2015). The impact of emotions on body-Focused repetitive behaviors: evidence from a non-treatment-seeking sample. *J Behav Ther Exp Psychiatry*, *46*, 189-197. https://doi.org/10.1016/j.jbtep.2014.10.007

Robinson, J. L., & Demaree, H. A. (2007). Physiological and cognitive effects of expressive dissonance. *Brain Cogn*, *63*(1), 70-78. https://doi.org/10.1016/j.bandc.2006.08.003

Robinson, M. D., & Clore, G. L. (2002). Belief and feeling: evidence for an accessibility model of emotional self-report. *Psychol Bull*, *128*(6), 934-960. https://doi.org/10.1037/0033-2909.128.6.934

Rodell, J. B., & Judge, T. A. (2009). Can "good" stressors spark "bad" behaviors? The mediating role of emotions in links of challenge and hindrance stressors with citizenship and counterproductive behaviors. *J Appl Psychol*, *94*(6), 1438-1451. https://doi.org/10.1037/a0016752

Ryan, R. M., & Deci, E. L. (2000). Self-determination theory and the facilitation of intrinsic motivation, social development, and well-being. *Am Psychol*, *55*(1), 68-78. https://doi.org/10.1037//0003-066x.55.1.68

Sayre, G. M., Grandey, A. A., & Chi, N. W. (2019). From cheery to "cheers"? Regulating emotions at work and alcohol consumption after work. *J Appl Psychol*. https://doi.org/10.1037/apl0000452

Schachter, S., & Singer, J. E. (1962). Cognitive, social, and physiological determinants of emotional state. *Psychol Rev*, *69*, 379-399. https://doi.org/10.1037/h0046234

Scott, B. A., & Barnes, C. M. (2011). A Multilevel Field Investigation of Emotional Labor, Affect, Work Withdrawal, and Gender. *ACADEMY OF MANAGEMENT JOURNAL*, *54*(1), 116-136. https://doi.org/Doi 10.5465/Amj.2011.59215086

Sheldon, K. M., & Elliot, A. J. (1999). Goal striving, need satisfaction, and longitudinal well-being: the self-concordance model. *J Pers Soc Psychol*, *76*(3), 482-497. https://doi.org/10.1037//0022-3514.76.3.482

Shepherd, B. R., Fritz, C., Hammer, L. B., Guros, F., & Meier, D. (2019). Emotional demands and alcohol use in corrections: A moderated mediation model. *J Occup Health Psychol*, *24*(4), 438-449. https://doi.org/10.1037/ocp0000114

Swann, W. B. (2005). The self and identity negotiation. *Interaction Studies*, *6*(1), 69-83. https://doi.org/https://doi.org/10.1075/is.6.1.06swa

Swann, W. B., & Read, S. J. (1981). Self-Verification Processes - How We Sustain Our Self-Conceptions. *Journal of Experimental Social Psychology*, *17*(4), 351-372. https://doi.org/Doi 10.1016/0022-1031(81)90043-3

Taris, T. W., & Feij, J. A. (2004). Learning and strain among newcomers: a three-wave study on the effects of job demands and job control. *J Psychol*, *138*(6), 543-563. https://doi.org/10.3200/JRLP.138.6.543-563

Thoresen, C. J., Kaplan, S. A., Barsky, A. P., Warren, C. R., & de Chermont, K. (2003). The affective underpinnings of job perceptions and attitudes: a meta-analytic review and integration. *Psychol Bull*, *129*(6), 914-945. https://doi.org/10.1037/0033-2909.129.6.914

Tomaka, J., Blascovich, J., Kelsey, R. M., & Leitten, C. L. (1993). Subjective, Physiological, and Behavioral-Effects of Threat and Challenge Appraisal. *Journal of Personality and Social Psychology*, *65*(2), 248-260. https://doi.org/Doi 10.1037/0022-3514.65.2.248

Totterdell, P., & Holman, D. (2003). Emotion regulation in customer service roles: testing a model of emotional labor. *J Occup Health Psychol*, *8*(1), 55-73. https://doi.org/10.1037//1076-8998.8.1.55

Vahtera, J., Kivimaki, M., Hublin, C., Korkeila, K., Suominen, S., Paunio, T., & Koskenvuo, M. (2007). Liability to anxiety and severe life events as predictors of new-onset sleep disturbances. *Sleep*, *30*(11), 1537-1546. https://doi.org/10.1093/sleep/30.11.1537

Wagner, D. T., Barnes, C. M., & Scott, B. A. (2014). Driving It Home: How Workplace Emotional Labor Harms Employee Home Life. *PERSONNEL PSYCHOLOGY*, *67*(2), 487-516. https://doi.org/10.1111/peps.12044

Watson, D. (2000). *Mood and temperament*. The Guilford Predd.

Wegner, D. M. (1994). Ironic processes of mental control. *Psychol Rev*, *101*(1), 34-52. https://doi.org/10.1037/0033-295x.101.1.34

Xanthopoulou, D., Bakker, A. B., Oerlemans, W. G. M., & Koszucka, M. (2018). Need for recovery after emotional labor: Differential effects of daily deep and surface acting. *Journal of Organizational Behavior*, *39*(4), 481-494. https://doi.org/10.1002/job.2245

Xiao, Z. M., Lee, M. H., & Wang, H. C. (2019). Service innovation and mental health: The multilevel moderating role of group emotional contagion. *SOCIAL BEHAVIOR AND PERSONALITY*, *47*(10). https://doi.org/10.2224/sbp.8143

Yanchus, N. J., Eby, L. T., Lance, C. E., & Drollinger, S. (2010). The impact of emotional labor on work-family outcomes. *JOURNAL OF VOCATIONAL BEHAVIOR*, *76*(1), 105-117. https://doi.org/10.1016/j.jvb.2009.05.001

Zapf, D., Vogt, C., Seifert, C., Mertini, H., & Isic, A. (1999). Emotion Work as a Source of Stress: The Concept and Development of an Instrument. *European Journal of Work and Organizational Psychology*, *8*(3), 371-400. https://doi.org/10.1080/135943299398230

**Table S3**

*Measures of Variables and Reliability Values*

| Variable | Paper | Measure | Reliability |
| --- | --- | --- | --- |
| Affect Spin | Beal et al. (2013)  (measured between-individuals) | Within-individual PA and NA were measured using the terms “enjoyment”, “happiness”, “anger”, “frustration”, “guilt” and “unhappiness”. Participants reported the extent to which they had experienced these states since the previous survey (“So far today” for the first survey) on a 5-point Likert scale (not at all to extremely). Then, within-individual-person-mean-centered scores were calculated for PA and NA and then transformed into the circular standard deviation of the affect circumplex. | PA α = .85  NA α = .77 |
| Affective experiences | Chang & Taxer (2021)  (measured within-individuals) | Three negative discrete emotions were assessed: anger, frustration, disappointment. (Keller et al., 2014). |  |
| Alcohol consumption | Sayre et al. (2019) (Study 1)  (measured within-individuals) | During the afternoon survey (between 1 and 5 p.m.) participants were asked how many standard alcoholic drinks (12 oz. of beer, 4 oz. of wine, 1 oz. of liquor) they consumed the prior day. |  |
|  | Sayre et al. (2019) (Study 2)  (measured within-individuals) | Alcohol consumption was measured the next morning (9 a.m.) as the number of standard alcoholic drinks (12 oz. of beer, 4 oz. of wine, 1 oz. of liquor) participants consumed since leaving work the prior day. |  |
| Anger | Keller et al. (2014),  (measured within-individuals and between-individuals) | Multiple times daily, a single item; i.e. “At the moment, how strongly do you experience anger?”, assessed anger on a 5-point Likert scale (not at all to very strongly). |  |
|  | Chang & Taxer (2021)  (measured within-individuals) | The respective items (for anger, frustration, disappointment) were formulated as follows: ‘At the moment, how strongly do you experience [the following emotion or affective experiences]?’, and were rated on a five-point scale from 1 (not at all) to 5 (strongly). |  |
| Anxiety | Keller et al. (2014)  (measured within-individuals and between-individuals) | Multiple times daily, a single item; i.e. “At the moment, how strongly do you experience anxiety?”, assessed enjoyment on a 5-point Likert scale (not at all to very strongly). |  |
|  | Wagner et al. (2014)  (measured within-individuals and between-individuals) | In the afternoon, participants indicated the extent to which they felt each of four adjectives (nervous, distressed, scared, and afraid; based on Mackinnon et al. (1999) and Watson et al. (1988)) at the moment using a 5-point Likert scale (very slightly or not at all to very much). | ᾱ = .73 |
| Felt Authenticity | Huppertz et al. (2020)  (measured within-individuals) | Felt authenticity was measured in the end of-work survey by three items taken from Erickson and Ritter’s (2001) Inauthenticity Scale. Responses were provided on a 5-point Likert scale ranging from 1 (never) to 5 (very often). Respondents were asked to think back about their interactions with customers “during today’s working day” when answering questions. An example item is “Today, I didn’t feel like I could be myself at work.” | ᾱ = .87 |
| Customer Conflict Handling | Huang et al. (2015)  (measured within-individuals) | At the end of the work day, 3 items by Ndubisi, Nelson, Oly et al. (2013) measured the extent to which agents tried to “openly discuss solutions when problems arise”, “solve conflicts before they occur” and “avoid potential conflicts with customers” during the day. | ᾱ = .79 |
| Deep Acting | Huppertz et al. (2020)  (measured within-individuals) | Deep acting was measured in the midday survey by the three items of Brotheridge and Lee’s (1998, 2003) Emotional Labor Survey. Responses were provided on a 5-point Likert scale ranging from 1 (I fully disagree) to 5 (I fully agree). When answering questions, respondents were asked to think about interactions with customers that morning. An example item is “Today, I made an effort to actually feel the emotions that I need to display to others.” | ᾱ = .77 |
|  | Huang et al. (2015)  (measured within-individuals) | In the middle of the work day, three items by Brotheridge and Lee (2003) reported how often participants engaged in actions such as “Make an effort to actually feel the emotions that you needed to display to others” during the morning, on a 5-point Likert scale (never to always). | ᾱ = .94 |
|  | Judge et al. (2009)  (measured within-individuals) | At the end of the work day, the three-item subscale from Brotheridge and Lee’s (1998) EL Scale was used to indicated how often participants had engaged in each of the activities on that day at work, i.e. “make an effort to actually feel the emotions that you needed to display to others”, “try to actually experience the emotions that you must show” and “really try to feel the emotions you have to show as part of your job”. Participants answered on a 5-point Likert scale (never to always). | ᾱ = .91 |
|  | Sayre et al. (2019) (Study 1)  (measured within-individuals) | In the afternoon (between 1 and 5 p-m.), three items by Brotheridge and Lee (2003) were used by participants to indicate on a 5-point Likert scale (never to always) how often they engaged in the following behaviors at work; e.g. “I tried to actually experience the emotions that I had to show”. | ᾱ = .88 |
| Deep Acting | Sayre et al. (2019) (Study 2)  (measured within-individuals) | DA was assessed in the evening (5 p.m.) using the same scale as in study 1. | ᾱ = .92 |
|  | Scott and Barnes (2011)  (measured within-individuals) | At the end of the work day, participants used the three-item scale by Grandey (2003) to indicate how often that day they had engaged in the actions listed, e.g. “Tried to actually experience the emotions I must show” and “Made an effort to actually feel the emotions that I needed to display toward others”. Answers were given on a 5-point Likert scale (never to always). | ᾱ = .94 |
|  | Totterdell and Holman (2003)  (measured within-individuals and between-individuals) | Measured within-individuals four times a day (11 a.m., 1 p.m., 3 p.m., and 5 p.m.) two items regarding positive refocus (which is a form of attentional deployment) and perspective taking (which is a form of cognitive change) asked participants to what extent they had, in respective order, “thought about pleasant things” and “thought about how customers feel,” (derived from time-sampling studies of ER strategies (Totterdell & Parkinson, 1999)). Participants answered on unipolar rating scales labeled 0 at one end to represent no extent and + (plus) at the other end to represent maximum extent and could select 1 of 19 possible response points along the rating scale.  Measured between-individuals, a questionnaire at the start of the study using the three-item scale by Brotheridge and Lee (1998) asked about participants DA, e.g. “How often do you try to actually experience the emotions you display to customers?”, with answers being given on a 5-point Likert scale (never to always). | Trait α = .85 |
| Depletion | Sayre et al. (2019) (Study 1)  (measured within-individuals) | In the evening (between 5 and 9 p-m.) the five-item scale based on Twenge et al. (2004) and published by Christian and Ellis (2011) asked about participants’ depletion by asking about the extent to which they agreed or disagreed with various statements, e.g. “I feel like my willpower is gone” on a 5-point Likert scale (strongly disagree to strongly agree). | ᾱ = .95 |
| Depletion | Sayre et al. (2019) (Study 2)  (measured within-individuals) | In the evening (5 p.m.) 10 items from Ciarocco et al. (2010) including items about a lack of energy like Study 1 (e.g., “I feel lazy”) plus temptation (e.g., “If I were tempted by something right now, it would be very difficult to resist”) asked about participants’ daily within-individual regulatory depletion. | ᾱ = .93 |
| Disappointment | Chang & Taxer (2021)  (measured within-individuals) | The respective items (for anger, frustration, disappointment) were formulated as follows: ‘At the moment, how strongly do you experience [the following emotion or affective experiences]?’, and were rated on a five-point scale from 1 (not at all) to 5 (strongly). |  |
| Situational Cue: Emotional Events | Totterdell and Holman (2003)  (measured within-individuals) | Measured within-individuals four times a day (11 a.m., 1 p.m., 3 p.m., and 5 p.m.), two scales, one for customers and one for coworkers, asked participants to what extent customers and coworkers had been pleasant on a response. Answers were given on two bipolar rating scales from unpleasant to pleasant with 19 response points. |  |
| Emotional Events (disruptive classroom event) | Chang & Taxer (2021) | The ESM survey contained two parts: (1) items asking teachers to identify the time the survey was taken (beginning of the day, mid-day planning period or lunch time, and end of the day), and if they had experienced a disruptive classroom event prior to responding to the survey. |  |
| Emotional Exhaustion | Huang et al. (2015)  (measured within-individuals) | In the middle of the work day, participants indicated their present degree of exhaustion on a 5-point Likert scale (never to always) regarding six items from the Shirom-Melamed Burnout Measure (Shirom & Melamed, 2006), e.g. “Feel emotionally drained from my work”. | ᾱ = .94 |
|  | Keller et al. (2014)  (measured between-individuals) | Measured by a paper-and-pencil questionnaire including nine items of the respective subscale of the Maslach Burnout Inventory (Maslach, Jackson, & Schwab, 1996) which was translated into German by Enzmann and Kleiber (1989), e.g. “I feel fatigued when I get up in the morning and have to face another day at school” on a 5-point Likert scale (not true at all to completely true) | α = .87 |
|  | Judge et al. (2009)  (measured within-individuals) | At the end of the work day, nine items (six from the Maslach Burnout Inventory (Maslach & Jackson, 1986) and three from the Shirom-Melamed Burnout Measure ((Shirom & Melamed, 2006)) asked the participants how often they felt each of the items that day e.g. “feel emotionally drained from your work”, “feel not capable of investing emotionally in coworkers and customers” and “feel that working with people all day is a strain for you” on a 5-point Likert scale (never to always). | ᾱ = .95 |
|  | Huppertz et al. (2020)  (measured within-individuals) | Emotional exhaustion was measured in the bedtime survey with two items from the Maslach Burnout  Inventory (Maslach & Jackson, 1981). We followed Teuchmann, Totterdell, and Parker (1999) and adapted the items to fit the daily setting. Participants were asked to indicate on a 5-point Likert scale how “emotionally drained” and “emotionally numb” they felt that day after work. Answers were provided on a 5-point Likert scale ranging from 1 (very slightly or not at all) to 5 (extremely). | ᾱ = .80 |
|  | Wagner et al. (2014)  (measured within-individuals) | In the evening, a paper-based survey was conducted prior to retiring to bed with four items from the emotional exhaustion scale (Maslach & Jackson, 1981). Participants indicated the extent to which they felt each of the statements at that moment, e.g. “Right now, I feel used up” and “Right now, I feel like I’m at the end of my rope” on a 5-point Likert scale (very slightly or not at all to very much). | ᾱ = .88 |
| Emotional Exhaustion | Totterdell and Holman (2003)  (measured within-individuals) | Assessed four times a day (11 a.m., 1 p.m., 3 p.m., and 5 p.m.) by the emotional exhaustion dimension of experienced burnout (Maslach & Jackson, 1981) and by emotional estrangement (Hochschild, 1983) which asked the extent to which participants “felt emotionally drained” and “felt emotionally numb” on two unipolar rating scales labeled 0 at one end to represent no extent and + (plus) at the other end to represent maximum extent with 19 possible response points. |  |
|  | Chang & Taxer (2021) (measured within-individuals) | We assessed teachers’ levels of emotional exhaustion, enjoyment in teaching, and feelings of being challenged by teaching. The respective items were formulated as follows: ‘At the moment, how strongly do you experience [the following emotion or affective experiences]?’, and were rated on a five-point scale from 1 (not at all) to 5 (strongly). |  |
| Emotional Expressivity | Totterdell and Holman (2003)  (measured between-individuals) | A questionnaire at the start of the study including four items of positive expressivity by Gross and John (1997) assessed between-individual emotional expressivity, e.g. “When I’m happy, my feelings show.”, assessed participants’ emotional expressivity on a 7-point Likert scale (strongly disagree to strongly agree). | α = .78 |
| Emotional Intelligence | Totterdell and Holman (2003)  (measured between-individuals) | Emotional intelligence was measured between individuals by a questionnaire at the start of the study consisting of Schutte et al.’s (1998) 33-item measure of emotional intelligence based on the model of emotional intelligence developed by Salovey and Mayer (1990), e.g. “I can tell how other people are feeling by listening to the tone of their voice.”. Participants answered on a 5-point Likert scale (strongly disagree to strongly agree). | α = .89 |
| Emotion Regulation Strategies | Chang & Taxer (2021) (measured between-individuals) | In the pre-survey, ten Likert-type scale items were adopted from Chang (2013) and Gross  and John (2003) to measure two emotion regulation strategies: reappraisal (4 items) and suppression (6 items). A sample item for reappraisal includes ‘I control my emotions by changing the way I think about the situation I’m in’. A sample item for suppression includes ‘I keep my emotions to myself in the classroom’. Participants responded to these items by indicating the extent to which they agreed to the statements on a 6-point scale from 1 (strongly disagree) to 6 (strongly agree). | reappraisal ⍺ = .81  suppression ⍺ = .75 |
| Emotional Job Demands | Sayre et al. (2019) (2)  (measured between-individuals) | Measured on the initial survey, three items by van de Ven et al. (2008) were used to assess between-individual emotional job demands which asked the participants the extent to which they agreed with the following statements: “I have to deal with people that get easily angered towards me”, “I have to do a lot of emotionally draining work”, “I have to deal with people (e.g. clients, colleagues or supervisors) whose problems touch me emotionally”. | α = .84 |
| Enjoyment | Keller et al. (2014)  (measured within-individuals) | Multiple times daily, a single item; i.e. “At the moment, how strongly do you experience enjoyment?”, assessed enjoyment on a 5-point Likert scale (not at all to very strongly). |  |
|  | Chang & Taxer (2021) (measured within-individuals) | We assessed teachers’ levels of emotional exhaustion, enjoyment in teaching, and feelings of being challenged by teaching. The respective items were formulated as follows: ‘At the moment, how strongly do you experience [the following emotion or affective experiences]?’, and were rated on a five-point scale from 1 (not at all) to 5 (strongly). |  |
| Expressed and Experienced Emotion | Totterdell and Holman (2003)  (measured within-individuals) | Measured within-individuals four times a day (11 a.m., 1 p.m., 3 p.m., and 5 p.m.), two rating scales assessed the pleasure and positive affect dimensions of the circumplex model of affect (Remington, Fabrigar, & Visser, 2000). Participants indicated to what extent they had displayed particular emotions and experienced those emotions (used in a previous time-sampling study (Totterdell, 1999)) using two bipolar rating scales, i.e. unhappy–happy and bored–enthused. |  |
| Extraversion | Judge et al. (2009)  (measured between-individuals) | Once during the course of the study, significant others were instructed to indicate how accurately several adjectives described the participant with regards to between-individual extraversion using Saucier’s Mini-Markers (1994). Items included “bold,” “talkative,” “bashful” (reverse scored), “extraverted,” and “quiet” (reverse scored). Answers were given on a 5-point Likert scale (extremely inaccurate to extremely accurate). | α = .70 |
| Fatigue | Beal et al. (2013)  (measured within-individuals) | Two items assessed fatigue on a 5-point Likert scale (strongly disagree to strongly agree), i.e. “Right now, I feel drained/exhausted” and “Right now, I feel very energetic” (reverse scored). The items were combined to form the fatigue measure (r = .61). | α = .76 |
| Felt Challenge | Huang et al. (2015)  (measured within-individuals) | In the middle of the work day, four items by Tomaka et al. (1993) and Boswell et al. (2004) assessed within-individual felt challenge. Participants were to what extent their tasks were seen as challenging during the first part of the day, e.g., “I view my tasks as challenging”. | ᾱ = .84 |
|  | Chang & Taxer (2021) (measured within-individuals) | We assessed teachers’ levels of emotional exhaustion, enjoyment in teaching, and feelings of being challenged by teaching. The respective items were formulated as follows: ‘At the moment, how strongly do you experience [the following emotion or affective experiences]?’, and were rated on a five-point scale from 1 (not at all) to 5 (strongly). |  |
| Frustration | Chang & Taxer (2021) (measured within-individuals) | The respective items (for anger, frustration, disappointment) were formulated as follows: ‘At the moment, how strongly do you experience [the following emotion or affective experiences]?’, and were rated on a five-point scale from 1 (not at all) to 5 (strongly). |  |
| Insomnia | Wagner et al. (2014)  (measured within-individuals) | Measured within-individuals prior beginning the work shift, four-items by Jenkins and colleagues (Jenkins, Jono, & Stanton, 1996; Jenkins, Stanton, Niemcryk, & Rose, 1988) asked participants to report how many hours they had slept and the extent to which they experienced various symptoms the prior night, e.g. “Woke up after your usual amount of sleep feeling tired and worn out” and “Woke up several times during the night”. Participants answered on a 5-point Likert scale (very slightly or not at all to very much). | ᾱ = .79 |
| Job Autonomy | Totterdell and Holman (2003)  (measured between-individuals) | A questionnaire at the start of the study consisting of adapted versions of Jackson et al.’s (1993) scales assessed two aspects of job autonomy, namely timing control and method control. Between-individual Timing control was measured by a five-item scale, e.g. “Can you vary how long you spend with a customer?” and between-individual method control was measured by a five-item scale, e.g. “Can you vary how you talk with customers?”. Both scales used a 5-point Likert scale (not at all to a great deal). | Timing Control α = .72; Method Control α = .69 |
| Job Satisfaction | Judge et al. (2009)  (measured within-individuals) | At the end of the work day, a five-item version of the Brayfield and Rothe (1951) Scale was used by participants to indicate the degree to which, on that day, they felt satisfied with their job, e.g. “felt enthusiastic about my work” and “the day at work seems like it will never end” (reverse scored) on a 5-point Likert scale (strongly disagree to strongly agree) . | ᾱ = .82 |
|  | Huang et al. (2015)  (measured within-individuals) | In the middle of the work day, participants indicated their state job satisfaction using three items by Cammann et al. (1979), e.g. “At present, I am satisfied with my job”. | ᾱ = .95 |
| Job Satisfaction | Bono et al. (2007)  (measured within-individuals and between-individuals) | Measured within-individuals, participants rated their agreement with the statement “At this very moment, I am fairly satisfied with my job.” on a 5-point Likert scale (strongly disagree to strongly agree).  Measured between-individuals, five items by Brayfield and Rothe (1951) were used for assessing general job satisfaction, e.g. “Most days I am enthusiastic about my work.” on a 5-point Likert scale (strongly disagree to strongly agree). |  |
| Mental Health | Xiao et al. (2019)  (measured within-individuals) | Participants’ mental health was assessed on a 5-point Likert scale (strongly disagree to strongly agree) using the 12-item scale of the Chinese Occupational Stress Index Lu et al. (1999) including three subscales measuring contentment, resilience, and peace of mind, e.g. “It seems like I cannot get the recognition that I deserve.” | α = .81 |
| Motive to Detach | Sayre et al. (2019) (Study 2)  (measured within-individuals) | In the evening (5 p.m.) participants completed the two-item scale adapted from Sonnentag and Fritz (2007) to assess within-individual motive to detach from work, i.e. “I want to forget about work” and “I need to detach from work”. | ᾱ = .80 |
| Negative Affect | Huppertz et al. (2020)  (measured within-individuals) | Positive and negative affect during work were measured in the midday survey with a measure developed by To, Fisher, Ashkanasy, and Rowe (2012). Respondents were asked to indicate on a 5-point Likert scale ranging from 1 (very slightly) to 5 (extremely) how they felt this morning. Negative affect was measured with four items (anxious, upset, ashamed, angry). | ᾱ = .86 |
|  | Judge et al. (2009)  (measured within-individuals and between-individuals) | Measured within-individuals using the Positive and Negative Affect Schedule—Expanded Form (PANAS-X; Watson et al., 1988), participants indicated the extent to which they felt each of the 10 items at the end of the work day reflecting the general dimension scale of NA “at work at that moment.”. Items included “sad”, “scornful”, “nervous”, “stressed” and “irritable”.  Measured between-individuals, each participant’s daily reports of NA were averaged. Thus, each daily report of PA and NA becomes an item in the scale. | State ᾱ = .95; Trait α = .92 |
|  | Sayre et al. (2019) (Study 1)  (measured within-individuals) | Measured within-individuals, in the evening (between 5 and 9 p.m.) seven items from the modified version of the PANAS (Watson et al., 1988) with items such as “irritable”, “jittery” and “tense” assessed participants’ state NA on a 5-point Likert scale (very slightly or not at all to very much). | ᾱ = .93 |
|  | Sayre et al. (2019) (Study 2)  (measured within-individuals) | The same scale was used as in study 1 but the assessment took place in the evening (5 p.m.). | ᾱ = .85 |
|  | Scott and Barnes (2011)  (measured within-individuals) | Measured within-individuals at the beginning and end of the work day with aid of the ten-item NA scale from the Positive and Negative Affect Scale (PANAS; Watson et al., 1988), participants indicated the extent to which they experienced each of the 10 items at work that day, e.g. “irritable”, “distressed” and “upset” on a 5-point Likert scale (very slightly or not at all to very much). | Beginning-of-work survey ᾱ = .72; end-of-work survey ᾱ = .70 |
| Negative Group Emotional Contagion | Xiao et al. (2019)  (measured between-individuals) | Nine items of the Emotional Contagion Scale (Doherty, 1997) measured the three subdimensions of anger, fear, and sadness between individuals on a 5-point Likert scale (strongly disagree to strongly agree), e.g. “It irritates me to be around angry people.” | α = .92 |
| Positive Affect | Huppertz et al. (2020)  (measured within-individuals) | Positive and negative affect during work were measured in the midday survey with a measure developed by To, Fisher, Ashkanasy, and Rowe (2012). Respondents were asked to indicate on a 5-point Likert scale ranging from 1 (very slightly) to 5 (extremely) how they felt this morning. Positive affect was measured with four items (excited, enthusiastic, interested, inspired). | ᾱ = .92 |
|  | Judge et al. (2009)  (measured within-individuals and between-individuals) | Measured within-individuals with the Positive and Negative Affect Schedule—Expanded Form (PANAS-X; Watson et al., 1988), participants indicated the extent to which they felt each of the 10 items reflecting the general dimension scale of PA at the end of the work day. They indicated how they felt “at work at that moment”, e.g. “excited”, “alert”, “cheerful”, “determined”, and “happy”.  Assessed between-individuals by averaging each participant’s daily reports of (measured within-individuals) PA. Thus, each daily report of PA and NA becomes an item in the scale. | State ᾱ = .96; trait  α = .93 |
|  | Scott and Barnes (2011)  (measured within-individuals) | At the beginning and end of the work day, the ten-item PA scale from Positive and Negative Affect Scale (PANAS; Watson et al., 1988) was used by participants to indicate the extent to which they were experiencing each emotion at the beginning and end of the work shift, e.g. “enthusiastic,” “excited,” and “attentive” on a 5-point Likert scale (very slightly or not at all to very much). | Beginning-of-work survey ᾱ = .95; end-of-work survey ᾱ = .95 |
|  | Totterdell and Holman (2003)  (measured within-individuals) | Two bipolar rating scales (unhappy–happy and bored–enthused) measured the pleasure and positive affect dimensions of the circumplex model of affect (Remington et al., 2000). Participants indicated to what extent they had displayed particular emotions and experienced those emotions (used in a previous time-sampling study by Totterdell (1999)). |  |
| Positive Group Emotional Contagion | Xiao et al. (2019)  (measured between-individuals) | Six items of the Emotional Contagion Scale (Doherty, 1997) measured the two subdimensions of love and happiness between individuals on a 5-point Likert scale (strongly disagree to strongly agree), e.g. “Being around happy people fills my mind with happy thoughts.” | α = .93 |
| Psychological Effort | Huppertz et al. (2020)  (measured within-individuals) | Psychological effort was measured in the end-of-work survey by the three items of the Explicit Emotional Effort Scale developed by Quiñones-García, Rodríguez-Carvajal, Clarke, and Moreno-Jiménez (2013). Responses were provided on a 5-point Likert scale ranging from 1 (never) to 5 (very often). Respondents were asked to think back when they engaged in the activity of meeting emotional display rules. An example item is “To what extent have you felt that the activity involved a great amount of effort?” | α = .91 |
| Rewarding Interactions | Huppertz et al. (2020)  (measured within-individuals) | Rewarding interactions were measured in the end-of-work survey by the four-item scale developed  by Brotheridge and Lee (2002). Responses were provided on a 5-point Likert scale ranging from 1 (never) to 5 (very often). Respondents were asked to think back about their interactions with customers “during today’s working day” when answering questions. The items were “Today, I put a lot more effort in my relationships with my clients than I got out of them,” “Today in my job, I ‘gave’ a lot but did not ‘get much’ in return,” “Today, I found my interactions with my clients unrewarding,” and “Today, I got very little thanks or recognition from my clients in return for my efforts.” | α = .81 |
| Service Innovation | Xiao et al. (2019)  (measured between-individuals) | At the end of the work day, the 16-item Service Innovation Questionnaire by Avlonitis et al. (2001) and Yen et al. (2012) was used by participants to evaluate their firm’s service innovation regarding the dimensions of radical service innovation (five items), incremental service innovation (four items), and strategic service innovation (seven items), e.g. “The service required the installation of new software by the company.”, “The service was a modification of an existing company product.” or “The service allowed the company to enter a new market for the first time.”. The used scale was a 5-point Likert scale (strongly disagree to strongly agree). | radical service innovation α = .90; strategic service innovation α = .84; incremental service innovation α = .84 |
| Service Performance | Totterdell and Holman (2003)  (measured within-individuals) | Measured within-individuals four times a day (11 a.m., 1 p.m., 3 p.m., and 5 p.m.), service performance was assessed consisting of job performance, proactivity, and expressed emotion (see measure Expressed and Experienced Emotion below). Job performance was measured using a bipolar rating scale (bad to good) that asked participants to rate the quality of their performance (adopted from a time-sampling study of mood and performance (Totterdell, 1999)). Proactivity was measured using a unipolar rating scale that asked participants to rate how much they had put themselves out to help customers (based on a measure used by Parker and Axtell (2001)). |  |
| Strain | Beal et al. (2013)  (measured within-individuals) | Strain was assessed by one single item: “Since the last survey (“So far today” for the first survey), to what extent have you experienced stress?”. Participants answered on a 5-point Likert scale (not at all to extremely). |  |
| Stress | Bono et al. (2007)  (measured within-individuals and between-individuals) | Measured within-individuals, participants responded to the statement “At this very moment, I am experiencing stress.” on a 5-point Likert scale (strongly disagree to strongly agree).  Measured between-individuals by a global measure of stress including four items by Motowidlo et al. (1986) using a 5-point Likert scale (strongly disagree to strongly agree). |  |
| Supervisor Support | Totterdell and Holman (2003)  (measured between-individuals) | A questionnaire at the start of the study including six items (based on a scale used by Parker et al. (1998)) assessed between-individual supervisor support, e.g. “Does your supervisor discuss and solve problems with you?”, assessed participants’ supervisor support on a 5-point Likert scale (not at all to a great deal). | α = .83 |
| Supervisors’ leadership behaviors | Bono et al. (2007)  (measured between-individuals) | Between-individual supervisors’ leadership behaviors were obtained from an organization-wide (N = 365) survey using the 20-item Multifactor Leadership Questionnaire (MLQ – Form 5x) by Avolio et al. (1995). Participants answered a 5-point Likert scale (not at all to frequently, if not always). Then, items were combined to form a single score by aggregating the leadership survey responses of all employees who completed a survey for the target supervisor (average n = 5 reports per supervisor, ICC-1 = .29 (p < .01) and ICC-2 = .72). |  |
| Surface Acting | Huppertz et al. (2020) | Surface acting was measured in the midday survey by the three items of Brotheridge and Lee’s (1998, 2003) Emotional Labor Survey. Responses were provided on 5-point Likert scale ranging from 1 (I fully disagree) to 5 (I fully agree). When answering questions, respondents were asked to think about interactions with customers that morning. An example item is “Today, I pretended to have emotions that I didn’t really have.” | ᾱ = .74 |
| Surface Acting | Beal et al. (2013)  (measured within-individuals) | Two items asked participants to indicate the frequency of hiding true emotions and expressing emotions that were different from their true emotions on a 5-point Likert scale (never to very often). Both items referred to the period since the servers had last completed a survey. The items were then averaged together as a measure of SA (r = .89). | **α = .94** |
|  | Bono et al. (2007)  (measured within-individuals and between-individuals) | Participants were asked about faking three positive emotions (happiness, enthusiasm, and optimism) and hiding three negative emotions (anxiety, irritation, and anger). They indicated their answers on a 5-point Likert scale (none at all to an intense amount).  Then, composite emotion variables were created, namely positive emotions faked (r = .93) and negative emotions hidden (r = .82). A total ER score was also formed by averaging the two types of ER (r = .88, p < .01). |  |
|  | Huang et al. (2015)  (measured within-individuals) | In the middle of the work day, three items by Brotheridge and Lee (2003) assessed how often the participants engaged in actions such as “Resist expressing your true feelings” during the morning, rated on a 5-point Likert scale (never to always). | ᾱ = .85 |
|  | Judge et al. (2009)  (measured within-individuals) | At the end of the work day, the three-item subscale from Brotheridge and Lee’s (1998) EL Scale: participants indicated how often they had engaged in each of the activities on that day at work, i.e. “resist expressing your true feelings”, “pretend to have emotions that you didn’t really have” and “hide your true feelings about a situation” on a 5-point Likert scale (never to always). | ᾱ = .91 |
|  | Keller et al. (2014)  (measured within-individuals and between-individuals) | Measured within-individuals, two items from the between EL scale were used, adapted to suit the momentary assessment, i.e. “At the moment I have to suppress my feelings.” and “At the moment, I have to display emotions that do not correspond to my inner feelings.” representing the two SA strategies suppression and faking, respectively. The items were subsequently combined into an overall scale (r = 0.63, p < 0.001 for (measured within-individuals)-level and r = 0.86, p < 0.001 for between-level)  Measured between-individuals, five items from a modified measure of the Frankfurt Emotion Work Scale (Zapf et al., 1999) by Neubach and Schmidt (2006) were used. The items were adapted to match the target group of teachers (substituting “work” with “class” and “customers” with “students”); e.g. “How often do you have to show feelings in class that you do not really feel?”. Responses were given on a 5-point Likert scale (never to very often). | Trait α = .91 |
|  | Sayre et al. (2019) (Study 1)  (measured within-individuals) | In the afternoon (between 1 and 5 p-m.), three items by Brotheridge and Lee (2003) indicated how often participants engaged in the following behaviors at work today; e.g. “I just pretended to have the emotions I displayed” on a 5-point Likert scale (never to always) | ᾱ = .90 |
|  | Sayre et al. (2019) (Study 2)  (measured within-individuals) | SA was assessed in the evening (5 p.m.) using the same scale as in study 1. | ᾱ = .93 |
| Surface Acting | Scott and Barnes (2011)  (measured within-individuals) | At the end of the work day, the five-item scale by Grandey (2003) was used by participants indicating how often that day they had engaged in the actions listed, e.g. “Put on an act in order to deal with customers in an appropriate way.” and “Faked a good mood.” on a 5-point Likert scale (almost never to 5 very often). | **ᾱ = .97** |
|  | Totterdell and Holman (2003)  (measured within-individuals and between-individuals) | Measured within-individuals four times a day (11 a.m., 1 p.m., 3 p.m., and 5 p.m.) one item regarding faking emotions (which is a form of response modulation) asked participants to what extent they had “expressed feelings you did not feel.” (derived from time-sampling studies of ER strategies (Parkinson & Totterdell, 1999) and emotional faking (Mann, 1999)). Responses were given on a unipolar rating scale labeled 0 at one end to represent no extent and + (plus) at the other end to represent maximum extent. Participants could select 1 of 19 possible response points along the rating scale.  Measured between-individuals, a questionnaire at the start of the study using three-item scale by Brotheridge and Lee (1998) assessed SA on a 5-point Likert scale (not at all to a great deal), e.g. “In order to do your job effectively, how often do you fake a good mood?” | Trait α = .76 |
|  | Wagner et al. (2014)  (measured within-individuals) | Following their shift in the afternoon, five items by Brotheridge and Lee (2003) and Grandey (2003) were used by participants indicating the extent to which each of the five statements described their work that day, e.g. “Today, I put on an act in order to deal with customers in an appropriate way.” or “Today, I just pretended to have the emotions I needed to display on the job.” Participants answered on a 5-point Likert scale (very slightly or not at all to very much). | ᾱ = .94 |
|  | Xiao et al. (2019)  (measured within-individuals) | The five-item SA subscale from Grandey’s (2003) EL Scale assessed participants’ SA on a 5-point Likert scale (never to always), e.g. “I put on an act to deal with customers in an appropriate way.” or “I just pretend to have the emotions I need to display for my job.” | α = .91 |
|  | Chang & Taxer (2021) (measured within- and between-individuals) | To measure teachers’ state-reported response modulation strategies, four items from the emotion regulation scale (Chang, 2013; Gross & John, 2003) were adapted to suit the momentary assessment. Teachers were instructed to report how often they employed the response modulation strategies of **suppression and faking** (‘I had to suppress my emotions’, and ‘I had to display positive emotions even if I didn’t feel them.’). Two items were used to capture if teachers chose to freely express their emotions instead (‘I expressed positive emotions to my students’, ‘I expressed negative emotions to my students’). Items were rated on a five-point frequency scale ranging from 1 (never) to 5 (always). |  |
| Work Withdrawal | Scott and Barnes (2011)  (measured within-individuals) | Measured within-individuals at the end of each work day, participants answered four items of the psychological withdrawal scale developed by Lehman and Simpson (1992), i.e. “Thought about being absent”, “Put less effort into the job than you should have”, “Thought about leaving current job” and “Daydreamed” on a 5-point Likert scale (almost never to very often). | ᾱ = .78 |
| Work-to-family conflict | Wagner et al. (2014)  (measured within-individuals) | A paper-based survey was conducted consisting of the three-item measure of strain-based work interference with family (Carlson et al., 2000). Participants indicated the extent to which each of the statements described them that evening just prior retiring to bed, e.g. “When I got home from work, I was too frazzled to participate in family activities/responsibilities.” on a 5-point Likert scale (very slightly or not at all to very much). | ᾱ = .89 |

*Note*. Information was included partly by literal citations and shortened. Blank spaces indicate that the information was not reported in the studies. EL = Emotional Labor. ER = Emotion Regulation. SA = Surface Acting. DA = Deep Acting.

**Table S4**

*Summary of the results of the included studies*

| Study | | Results | | | | |
| --- | --- | --- | --- | --- | --- | --- |
| Beal et al. (2013) | | - Positive effect of SA on strain (a = .24, β_a_ = .17, *p* < .05) and positive effect of strain on fatigue (b = .28, β_b_ = .32, *p* < .05) - Indirect effect of SA on fatigue through strain (ab = .07, *p* < .05). - Direct effect of SA on fatigue over and above any indirect effect through strain ([c´] = .23, β_c’_ =.19, *p* < .05) - Cross-level moderating effect for path b (effect of strain on fatigue; γ = -.36, *p* < .05) with the simple slope for low-affect spin individuals being positive and significant (a = .12, β_α_ = .09, *p* < .05), but the simple slope for high-affect spin servers being stronger (a = .35, β_α_ = .26, *p* < .05) - Cross-level moderating effect for path c´ (direct effect of SA on fatigue controlling for strain; γ = .15, *p* < .05) with the simple slope for low-affect spin individuals being positive and significant (α = .12, β_α_ = .09, *p* < .05), but the simple slope for high-affect spin servers being stronger (α = .35, β_α_ = .26, *p* < .05) | | | | |
| Bono et al. (2007) | | - Positive effect of both types of SA (hiding negative and faking positive emotions) on stress (γ = .40 and .35, *p* < .01, for hiding negative and faking positive, respectively) - No moderating effect of supervisors’ transformational leadership behaviors on the stress – SA relationship (γ = .01, *p* < .01) - Negative effect of SA on job satisfaction (γ = –.18 and –.18, *p* < .01, for hiding negative and faking positive, respectively) - Moderating effect of transformational leadership (γ = .21, *p* < .01) on the SA – job satisfaction relationship: when transformational leadership behaviors were low (1 SD below the mean), episodes of SA were associated with decreased job satisfaction and when transformational leadership behaviors were high (1 SD above the mean), there was little or no association between SA and job satisfaction | | | | |
| Chang et al. (2021) | | - significant multivariate differences between low (LERG) and high habitual reappraisal (HRG) group, between low reappraisal (LERG) and high habitual suppression (HSRG) group, high suppression (HSRG) and high reappraisal (HRG) group, respectively in emotions reported following the classroom incidents (F (12, 120) = 4.34, p < .001; Wilk’s Λ = 0.49, partial η2 = .30). - Univariate results were:   - Anger: LERG vs. HRG; LERG vs. HSRG   - Emotional Exhaustion: HRG vs. HSRG   - Enjoyment: LERG vs. HRG; HRG vs. HSRG   - Feeling Challenged: HRG vs. HSRG - significant multivariate differences between low (LERG) and high habitual reappraisal (HRG) group, between low reappraisal (LERG) and high habitual suppression (HSRG) group, high suppression (HSRG) and high reappraisal (HRG) group, respectively in response modulation strategies (SA, DA) (F (10, 118) = 3.88, p < .001; Wilk’s Ë = 0.57, partial ç2 = .25) - Univariate results were:   - Expression of Positive Emotions to Students HRG vs. HSRG   - Suppress Emotions HRG vs. HSRG | | | | |
| Huang et al. (2015) | | - DA was negatively correlated with (*r* = -.11, p < .001) and had a negative effect (B = -.12, *p* < .001) on emotional exhaustion and negative effect of felt challenge on emotional exhaustion (B = -.06, p < .05) - Moderating effect of felt challenge on the DA – emotional exhaustion relationship (B = -.15, *p* < .001): employees were less exhausted when DA while feeling challenged - Negative effect of DA on job satisfaction (B = .15, *p* < .001) - Moderating effect of felt challenge on the DA – job satisfaction relationship: the effect was accentuated for higher felt challenge (B = .12, *p* < .01) - Mediating effect of emotional exhaustion between DA and emotional exhaustion: when adding emotional exhaustion to the model (emotional exhaustion to outcome, controlling for the predictors (DA, SA, felt challenge and DA – felt challenge interaction)), emotional exhaustion added significantly to the prediction (B = -.37, *p* < .01), supporting the condition for mediation. Monte Carlo analyses (Selig & Preacher, 2008) revealed DA influenced momentary job satisfaction through emotional exhaustion (indirect effect B_ab_ = .04, *p* < .001), and the DA – felt challenge interaction term was no longer significant after controlling for emotional exhaustion, indicating complete mediation. - Positive effect of DA on customer conflict handling (B = .10, *p* < .001) - Moderating effect of felt challenge on the DA – customer conflict handling relationship: felt challenge magnified DA’s positive relationship with customer conflict handling (B = .08, *p* < .01) - Mediating effect of emotional exhaustion between DA and conflict handling: when adding emotional exhaustion to the model (emotional exhaustion to outcome, controlling for the predictors (DA, SA, felt challenge and DA – felt challenge interaction)), emotional exhaustion added significantly to the prediction (B = -.09, *p* < .01), supporting the condition for mediation. Monte Carlo analyses (Selig & Preacher, 2008) revealed emotional exhaustion mediated the main effect of DA (B_ab_ = .01, *p* < .01) and the interaction of DA – felt challenge interaction on customer conflict handling (B_ab_ = .01, *p* < .01). The DA – felt challenge interaction term was no longer significant after controlling for emotional exhaustion, indicating complete mediation. | | | | |
| Hupperts et al.( 2020) | - SA was positively related to psychological effort (estimate .13, 95% CI [.04, .23]) > neu - DA was not significantly related to psychological effort (estimate .01, 95% CI [.11, .09]) > neu - the SA–psychological effort relationship was not significantly stronger than the DA–psychological effort relationship at the 95% confidence level (difference .15,95% CI [.01, .29]). > neu - SA was found to negatively relate to felt authenticity (estimate .15, 95% CI [.23, .07]) > neu - DA was not significantly related to felt authenticity (estimate .02, 95% CI [.12, .08]) > neu - SA was found to negatively relate to rewarding interactions (estimate .16, 95% CI [.24, .08]) > neu - DA was not significantly related to rewarding interactions (estimate .01, 95% CI [.09, .07]). > neu - Psychological effort (estimate .04, 95% CI [.01, .08]) and felt authenticity (estimate .04, 95% CI [.01, .09]) mediated the [positive] relationship between SA and emotional exhaustion. > SA-EE passt zu bisherigen Ergebnissen, Mediatoren sind neu - Rewarding interactions did not mediate the relationship between SA and emotional exhaustion (estimate .02, 95% CI [.05, .01]) > neu - None of the mechanisms—that is, psychological effort (estimate .01, 95% CI [.04, .02]), felt authenticity (estimate .00, 95% CI [.03, .02]), and rewarding interactions (estimate .00, 95% CI [.01, .01])—meditated the relationship between DA and emotional exhaustion. > neu - There was also no evidence for a total effect of DA and emotional exhaustion (estimate .04, 95% CI [.15, .08]). > kein DA-EE Zusammenhang past zu bisherigen Ergebnissen - DA was positively related to positive affect, which, in turn, was positively related to rewarding interactions and negatively to emotional exhaustion. > DA-PA passt (bisherige Ergebnisse waren bereits widersprüchlich) | |  |  |  |  |
| Judge et al. (2009) | - Positive effect of SA on NA (β = .354, *p* < .05) - Moderating effect of extraversion on the SA – NA relationship: extraversion did predict the slope of this relationship such that SA was associated with lower NA for extraverts and higher NA for introverts (β = −.055, *p* < .05) - Positive effect of SA on emotional exhaustion (β = .634; *p* < .01) - Moderating effect of extraversion on the SA – emotional exhaustion relationship: extraversion did predict the slope of this relationship, whereby the association was significantly stronger for introverts, meaning that SA was more emotionally exhausting for introverts than for extraverts (β = −.074, *p* < .01) - Negative effect of SA on job satisfaction (β = −.465, *p* < .05) - Mediating effect of state NA between SA and job satisfaction: NA mediated slightly less than half of the association (45.76%) - No moderating effect of extraversion on the SA – job satisfaction relationship (β = .044, ns) - Negative effect of DA on PA (β = −.435, *p* < .05) - Moderating effect of extraversion on the DA – PA relationship: DA was associated with lower levels of PA for introverts but for extraverts, DA was associated with higher levels of PA (β = .095, *p* < .01) - No effect of DA on job satisfaction (β = −.331, ns) either before or after controlling for PA - No moderating effect of extraversion on the DA – job satisfaction relationship (β = .086, ns) | | | | |  |
| Keller et al. ( 2014) | - Positive effect of trait SA on state SA (b = 0.22, *p* < 0.001) on the between-level - Positive effect of anger on SA (b = 0.88, *p* < 0.0001) on the between-level, even after controlling for trait emotional exhaustion and trait SA (b = 0.89, *p* < 0.0001) - Positive effect of anger on SA (b = 0.34, *p* < 0.001) on the within-level - Negative effect of enjoyment (b = -0.10, *p* < 0.01) and anxiety (b = 0.30, *p* < 0.01) on SA on the within-level - No effect of emotional exhaustion on state SA when controlling for trait SA on the between-level | | | | |  |
| Sayre et al. (2019) | - Study 1: No effect of SA on depletion after controlling for NA and DA (estimate = .04, SE = .11, *p* = .75, 95% CI [-.14, .21]) and no effect of depletion on alcohol consumption (estimate = .06, SE = .08, *p* = .48, 95% CI [-.08, .19]). No indirect effect of SA on alcohol use through depletion (estimate = .002, SE = .01, *p* = 95% CI [-.01, .01]) - Study 2: Positive effect of SA on depletion after controlling for NA and DA, (estimate = .13, SE = .06, *p* = .03, 95% CI [.03, .22]) but no effect of depletion on alcohol consumption beyond the covariates (estimate = -.01, SE = .04, *p* = .73, 95% CI [-.07, .05]). No indirect effect of SA on alcohol use through depletion (estimate = .01, SE = .01, 95% CI [-.01, .01]) - Study 2: No effect of SA on alcohol use after controlling for NA, DA, and depletion (estimate = -.13, SE = .11, *p* = .75, 95% CI [-.31, .05]) and SA did not significantly predict alcohol consumption beyond controls (estimate = .01, SE = .04, *p* = .82, 95% CI [-.05, .07]) - Study 2: No effect of SA on the motive to detach (estimate = .06, SE = .06, *p* = .32, 95% CI [-.05, .07]) - Study 2: No indirect effect for SA on alcohol use through the motive to detach (estimate = .02, SE = .01, 95% CI [-.01, .03]) - Study 2: Moderating effect of chronic emotional job demands on the SA – alcohol use relationship (estimate = .10, SE = .04, *p* = .01, 95% CI [.04, .16]): more SA than normal was positively related to daily alcohol use for those with high emotional demands (b = .13, SE = .05 t = 2.55, *p* = .01) and not significantly related to daily alcohol use for those with low emotional demands (b = -.10, SE = .06, t = -1.69, *p* = .09) - Study 2: No indirect effect of the interaction of SA and emotional job demands on alcohol use through depletion (95% CI [-.01, .01]) - Study 1: Negative effect of DA on alcohol use after controlling for NA and SA (estimate = -.23, SE = .11, *p* = .047, 95% CI [-.43, -.04]): On days when individuals deep act one scale unit more than normal, alcohol consumption was predicted to be .79 times lower (e_-.23_ = .79) - Study 1 and 2: No effect of DA on depletion (estimate = -.07, SE = .12, *p* = .58, 95% CI [-.27, .14] and estimate = -.03, SE = .06, p = .64, 95% CI [-.12, .07]) - Study 1: No indirect effect of DA on alcohol use through depletion (estimate = -.004, SE = .01, 95% CI [-.02, .01]) - Study 2: No indirect effect of DA on alcohol consumption through depletion (estimate = .001, SE = .01, 95% CI [-.01, .01]), even when controlling for the covariates (estimate = -.01, SE = .04, *p* = .73, 95% CI [-.07, .05]) - Study 2: Negative effect of DA on the motive to detach from work (estimate = -.10, SE = .04, *p* = .03, 95% CI [-.13, -.02]) - Study 2: Negative indirect effect of DA on alcohol use via the motive to detach (estimate = -.02, SE = .01, 95% CI [-.03, -.003]) - Study 2: No moderating effect of emotional job demands on the DA – alcohol use relationship (estimate = .03, SE = .04, *p* = .33, 95% CI [-.02, .09]) | | | |  |  |
| Wagner et al.(2014) | - Positive effect of SA on anxiety (B = .16, *p* < .01) - Positive effect of SA on emotional exhaustion from one day to the next (B = .23, *p* < .01). - No mediating effect of state anxiety between SA and emotional exhaustion: despite a significant indirect effect (b = .12, *p* < .05), a comparison of the main effect of SA on emotional exhaustion did not significantly change from Model 1 to Model 2 (*Z* = .19, *p* > .10). This suggests that even though SA had a significant effect on emotional exhaustion via anxiety, state anxiety did not explain the main effect of SA on emotional exhaustion. - Positive effect of SA on night-time insomnia (B = .19, *p* < .05) - Mediating effect of state anxiety between SA and insomnia (b = .07, *p* < .05): the effect of SA on insomnia was reduced by 37% when including anxiety as a predictor - Positive effect of SA on work-to-family conflict from one day to the next (B = .33, *p* < .01) - No mediating effect of state anxiety between SA and work-to-family conflict (b = .13, *p* = .075) | | |  |  |  |
| Xiao et al. (2019) | - Positive effect of service innovation on SA (γ = .318, *p* < .01) - Negative effect of SA on mental health (γ = -.482, *p* < .01) - Negative effect of negative group emotional contagion on mental health (γ = -.146, *p* < .05) - No moderating effect of negative group emotional contagion on the SA – mental health relationship (γ = -.014, ns). - Positive effect of positive group emotional contagion on mental health (γ = .517, *p* < .01) - Moderating effect of positive group emotional contagion on the SA – mental health relationship (γ = .227, *p* < .05): the negative effect of SA on mental health was lower when positive group emotional contagion was high and vice versa | | |  |  |  |
| Scott et al. (2011,  Study 1) | - Positive effect of SA on work withdrawal (b = 0.20, *p* < .05) and on state NA (b = 0.08, *p* < .05) - Moderating effect of gender on the SA – state NA relationship (b = 0.14, *p* < .05): the relationship was stronger for females than males with the relationship being significant for females (b = 0.16, z = 4.17, *p* < .05) but not males (b = - 0.03, *z* = - 1.29, ns) - No effect of SA on state PA (b = - 0.00, ns) and no moderating effect of gender on the SA – state PA relationship (b = - 0.01, ns) - Positive effect of state NA on employees’ reports of work withdrawal (b = 0.20, *p* < .05) - Mediating effect of NA between SA and work withdrawal (*z* = 2.20, *p* < .05): for females, results revealed a significant indirect effect of SA (*z* = 2.07, *p* < .05) on perceptions of work withdrawal via state NA. For males however, the indirect effect was not significant (*z* = 1.27, ns). Despite this difference, the confidence intervals for the indirect effect overlapped, thus precluding the conclusion that the mediated effect differed significantly by gender. - No effect of state PA on employees’ reports of work withdrawal (b = 0.02, ns) and no mediating effect of PA between SA and work withdrawal - Moderating effect of gender on the SA – work withdrawal relationship (b = 0.25, *p* < .05): the relationship was stronger for females than males. Simple slopes analyses revealed that the relationship between SA and perceptions of work withdrawal was significant for both females (b = 0.35, *z* = 5.07, *p* < .05) and males (b = 0.10, *z* = 2.07, *p* < .05). - No effect of DA on work withdrawal (b = -0.04, ns) and negative effect of DA on state NA (b = -0.06, *p* < .05) - Positive effect of State NA on employees’ reports of work withdrawal (b = 0.20, *p* < .05) - Moderating effect of gender on the DA – state NA relationship (b = - 0.11, *p* < .05): the relationship was stronger for females than males with the relationship being significant for females (b = - 0.14, *z* = 4.17, *p* < .05) but not males (b = - 0.03, *z* = - 1.29, ns). - Positive effect of DA on state PA (b = 0.12, *p* < .05) and no moderating effect of gender on the DA – state PA relationship | | |  |  |  |
| Scott et al. (2011,  Study 2) | - Mediating effect of NA between DA and work withdrawal (z = 2.81, *p* < .05): for females, results revealed a significant indirect effect of DA (z = 2.31, *p* < .05) on perceptions of work withdrawal via state NA. For males however, the indirect effect was not significant for DA (*z* = 1.43, ns). Despite these differences, the confidence intervals for the indirect effect overlapped, thus precluding the conclusion that the mediated effect differed significantly by gender. - No effect of state PA on employees’ reports of work withdrawal (b = 0.02, ns) and no mediating effect of PA between DA and perceptions of work withdrawal - Moderating effect of gender on the DA – perceptions of work withdrawal relationship (b = -0.14, *p* < .05): the relationship was stronger for females than males. The relationship between DA and perceptions of work withdrawal was significant for females (b = -0.14, *z* = 2.54, *p* < .05) but not males (b = 0.00, *z* = .03). | | |  |  |  |
| Totterdell et al., (2003) | - Positive effect of SA (faking emotions) on feeling emotionally drained and emotionally numb (.28, p < .01 and .08, p < .05 respectively) - No effect DA (positive refocus and perspective taking) on feeling emotionally drained (.04, ns and .06, ns) or emotionally numb (.01, ns and -.04, ns) - Positive effect of DA on the quality of performance (both .09, p < .01) and displayed enthusiasm (.06, p < .05 and .08, p < .01) - No effect of SA on the quality of performance (-.01, ns) and displayed enthusiasm (.01, ns) - Positive effect of positive refocus (DA) on displayed happiness (.10, p < .01) but not proactivity in helping customers (.13, ns) - Positive effect of perspective taking (.48, p < .01) and faking emotions (.19, p < .01) on proactivity - No effect of perspective taking (.03, ns) and faking (-.05, ns) on displayed happiness - Positive effect of positive events on DA (see below) - Moderating effect of event source on the event – EL relationship: the relationship of positive events with perspective taking (DA) was stronger when events emerged from customers rather than coworkers (.13, p < .05 and .14, ns respectively). Positive events from coworkers were more strongly associated with positive refocus than events from customers (.37, p < .01 and .29, p < .05 respectively). Negative (unpleasant) events from customers but not from coworkers were associated with faking emotions (SA) (- .18, *p* < .01 and .05, ns respectively). - No effect of the organizational variables of job autonomy and supervisor support on SA and DA - No effect of positive emotional expressivity on SA (-.08, ns) and DA (.26, ns and -.24, ns) and positive effect of emotional intelligence on positive refocus (.09, p < .05) but not on perspective taking (.05, ns), or faking emotions (.03, ns) - Moderating effect of gender on the antecedent – EL relationships: females engaged in higher levels of faking emotions (-1.90, p < .05), but did not differ from males in positive refocus (2.24, ns) and perspective taking (-1.21, ns) | | |  |  |  |

*Note.* Blank spaces indicate that the information was not reported in the studies. SA = Surface Acting. DA = Deep Acting. PA = Positive Affect. NA = Negative Affect
